# Bcl-xL acts as an inhibitor of IP_3_R channels, thereby antagonizing Ca^2+^-driven apoptosis

**DOI:** 10.1038/s41418-021-00894-w

**Published:** 2021-11-08

**Authors:** Nicolas Rosa, Hristina Ivanova, Larry E. Wagner, Justin Kale, Rita La Rovere, Kirsten Welkenhuyzen, Nikolaos Louros, Spyridoula Karamanou, Victoria Shabardina, Irma Lemmens, Elien Vandermarliere, Kozo Hamada, Hideaki Ando, Frederic Rousseau, Joost Schymkowitz, Jan Tavernier, Katsuhiko Mikoshiba, Anastassios Economou, David W. Andrews, Jan B. Parys, David I. Yule, Geert Bultynck

**Affiliations:** 1 KU Leuven, Laboratory of Molecular and Cellular Signaling, Department of Cellular and Molecular Medicine, and Leuven Kanker Instituut, Campus Gasthuisberg O/N-1 Box 802, Herestraat 49, 3000 Leuven, Belgium; 2 Department of Pharmacology and Physiology, School of Medicine and Dentistry, University of Rochester, 601 Elmwood Avenue, Box 711, Rochester, NY 14642 USA; 3 Biological Sciences, Sunnybrook Research Institute, University of Toronto, Toronto, ON M4N 3M5 Canada; 4 VIB Center for Brain and Disease Research, Campus Gasthuisberg O/N-1bis Box 802, Herestraat 49, 3000 Leuven, Belgium; 5 KU Leuven, Switch Laboratory, Department of Cellular and Molecular Medicine, Campus Gasthuisberg O/N-1bis Box 802, Herestraat 49, 3000 Leuven, Belgium; 6 KU Leuven, Laboratory of Molecular Bacteriology, Department of Microbiology and Immunology, Rega Institute for Medical Research, Campus Gasthuisberg P.O, Box 1037, Herestraat 49, 3000 Leuven, Belgium; 7 Institut of Evolutionary Biology, CSIC-Universitat Pompeu Fabra, Passeig Marítim de la Barceloneta 37-49, 08003 Barcelona, Spain; 8 Cytokine Receptor Laboratory, Faculty of Medicine and Health Sciences, Department of Biomolecular Medicine, Ghent University, and Center for Medical Biotechnology, VIB, Albert Baertsoenkaai 3, B-9000 Ghent, Belgium; 9 VIB-UGent Center for Medical Biotechnology, VIB, Ghent, 9000 Belgium; 10 Shanghai Institute for Advanced Immunochemical Studies, ShanghaiTech University, 201210 Shanghai, China; 11 Laboratory for Developmental Neurobiology, RIKEN Brain Science Institute, 2-1 Hirosawa, Wako, 351-0198 Saitama Japan; 12 Faculty of Science, Toho University, Miyama 2-2-1, Funabashi, 274-8510 Chiba Japan

**Keywords:** Cancer, Cell biology, Molecular biology

## Abstract

Anti-apoptotic Bcl-2-family members not only act at mitochondria but also at the endoplasmic reticulum, where they impact Ca^2+^ dynamics by controlling IP_3_ receptor (IP_3_R) function. Current models propose distinct roles for Bcl-2 *vs.* Bcl-xL, with Bcl-2 inhibiting IP_3_Rs and preventing pro-apoptotic Ca^2+^ release and Bcl-xL sensitizing IP_3_Rs to low [IP_3_] and promoting pro-survival Ca^2+^ oscillations. We here demonstrate that Bcl-xL too inhibits IP_3_R-mediated Ca^2+^ release by interacting with the same IP_3_R regions as Bcl-2. Via *in silico* superposition, we previously found that the residue K87 of Bcl-xL spatially resembled K17 of Bcl-2, a residue critical for Bcl-2’s IP_3_R-inhibitory properties. Mutagenesis of K87 in Bcl-xL impaired its binding to IP_3_R and abrogated Bcl-xL’s inhibitory effect on IP_3_Rs. Single-channel recordings demonstrate that purified Bcl-xL, but not Bcl-xL^K87D^, suppressed IP_3_R single-channel openings stimulated by sub-maximal and threshold [IP_3_]. Moreover, we demonstrate that Bcl-xL-mediated inhibition of IP_3_Rs contributes to its anti-apoptotic properties against Ca^2+^-driven apoptosis. Staurosporine (STS) elicits long-lasting Ca^2+^ elevations in wild-type but not in IP_3_R-knockout HeLa cells, sensitizing the former to STS treatment. Overexpression of Bcl-xL in wild-type HeLa cells suppressed STS-induced Ca^2+^ signals and cell death, while Bcl-xL^K87D^ was much less effective in doing so. In the absence of IP_3_Rs, Bcl-xL and Bcl-xL^K87D^ were equally effective in suppressing STS-induced cell death. Finally, we demonstrate that endogenous Bcl-xL also suppress IP_3_R activity in MDA-MB-231 breast cancer cells, whereby Bcl-xL knockdown augmented IP_3_R-mediated Ca^2+^ release and increased the sensitivity towards STS, without altering the ER Ca^2+^ content. Hence, this study challenges the current paradigm of divergent functions for Bcl-2 and Bcl-xL in Ca^2+^-signaling modulation and reveals that, similarly to Bcl-2, Bcl-xL inhibits IP_3_R-mediated Ca^2+^ release and IP_3_R-driven cell death. Our work further underpins that IP_3_R inhibition is an integral part of Bcl-xL’s anti-apoptotic function.

## Introduction

Inositol 1,4,5-trisphosphate receptors (IP_3_Rs) are tetrameric Ca^2+^-permeable channels, predominantly located at the endoplasmic reticulum (ER) membrane [1–3]. Ca^2+^ release through IP_3_Rs plays fundamental roles in a plethora of cellular processes, including proliferation, gene transcription, protein secretion, neurotransmitter release, fertilization, and apoptosis [4]. To maintain fidelity and specificity of these processes the activity of IP_3_Rs is tightly regulated at multiple levels. Among the most common regulatory mechanisms are the modulation of channel expression, post-translational modifications, and interaction with regulatory factors including Ca^2+^ itself, ATP and protein partners [1, 5, 6]. These regulators target different IP_3_R regions, which are arranged as globular domains such that the controlled trypsinization of IP_3_R generates five reproducible fragments [7], which have proven an excellent tool for dissecting the binding sites of different IP_3_R partners [8–12].

The B-cell lymphoma 2 (Bcl-2) family of proteins is well known for its role in controlling mitochondrial apoptosis and mitochondrial dynamics [13, 14]. Anti-apoptotic Bcl-2 family members neutralize pro-apoptotic family members, including Bax/Bak and pro-apoptotic BH3-only proteins [15]. At the molecular level, anti-apoptotic Bcl-2 family members use their hydrophobic cleft (formed by the BH3-BH1-BH2 domains) to bind the BH3 domains of pro-apoptotic Bcl-2 family proteins [16–18]. Bcl-2 proteins also act at the ER, where they impact Ca^2+^ homeostasis [19]. Anti-apoptotic Bcl-2 and B-cell lymphoma-extra large (Bcl-xL) have emerged as important IP_3_R modulators [20, 21]. The present consensus is that Bcl-2 inhibits the IP_3_R channel activity [20, 22]. At the molecular level, inhibition of channel activity prominently occurs through the BH4 domain of Bcl-2 (BH4-Bcl-2) that targets several regions of the IP_3_R channel. Initially, inhibition of IP_3_R by Bcl-2 was explained by the interaction between the BH4 domain of Bcl-2 and a stretch of 20 amino acids (a.a. 1389–1408 of mouse IP_3_R1) located in the central, modulatory region and more specifically in the third tryptic IP_3_R fragment (Fragment 3) [8, 23]. Recently, the BH4 domain was also found to target the ligand-binding domain (LBD), particularly the IP_3_-binding core (a.a. 226–604) [24]. In addition, we revealed a critical role for the C-terminal transmembrane domain of Bcl-2 to recruit the protein near the 6^th^ helix of the transmembrane domain of the IP_3_R [11, 12].

In contrast to Bcl-2, Bcl-xL has been suggested to sensitize IP_3_Rs [25], specifically to promote channel opening at lower concentrations of IP_3_. Indeed, in DT40 cells, Bcl-xL was reported to increase IP_3_ single-channel activity and to promote Ca^2+^ oscillations by sensitizing IP_3_Rs. This effect in turn was proposed to maintain cell survival by optimizing mitochondrial bio-energetics. Moreover, it was shown that this pro-survival function of Bcl-xL relies predominantly on IP_3_Rs, since Bcl-xL overexpression in DT40 cells lacking all three endogenous IP_3_R isoforms (3KO) was much less effective in protecting the cells against pro-apoptotic stimuli [25]. At the molecular level, Bcl-xL was proposed to act by targeting two BH3-like domains in the C-terminal part of the channel to account for IP_3_R sensitization [26]. In that study, Bcl-xL was reported to only weakly bind to the central, modulatory region, underlying inhibition of IP_3_Rs only at very high Bcl-xL concentrations.

We previously demonstrated a critical and unique role for the K17 residue in the BH4 domain of Bcl-2 to mediate the inhibition of IP_3_R activity. A lysine residue at this position is not present in the BH4 domain of Bcl-xL (BH4-Bcl-xL) and this domain fails to inhibit IP_3_R activity [27]. Notably, in the linear sequence of Bcl-2, K17 corresponds to D11 of Bcl-xL, and substitution of K17 to an aspartate residue in Bcl-2 abrogated the ability of the BH4 domain to inhibit IP_3_Rs. Conversely, switching D11 to a lysine residue in Bcl-xL rendered BH4-Bcl-xL capable of inhibiting IP_3_Rs. These data appear to provide a rationale for the distinct actions of Bcl-2 and Bcl-xL. Nevertheless, using structural modeling, we previously found that another positively-charged residue, K87, located in the BH3 domain of Bcl-xL, spatially resembled the K17 in the BH4-Bcl-2 [28]. This observation prompted us to revisit the idea that Bcl-xL is truly an IP_3_R-sensitizing protein. In contrast to existing literature about Bcl-xL, we found that Bcl-xL inhibits IP_3_R-mediated Ca^2+^ release, when overexpressed in living cells, and IP_3_R single-channel openings, when applied as purified protein. At the molecular level, we demonstrate that Bcl-xL binds to full-length IP_3_R, and could target the same IP_3_R regions as the one targeted by Bcl-2’s BH4 domain, namely the ligand-binding domain and the central, modulatory region. Moreover, mutating K87 to D in Bcl-xL impaired its ability to bind to full-length IP_3_Rs, as well as to the ligand-binding and the central, modulatory regions. In line with these observations, the mutant Bcl-xL^K87D^ failed to inhibit IP_3_R function in living cells and in single-channel recording. Furthermore, we show that K87 of Bcl-xL is critical to protect cells against staurosporine-induced apoptosis which is dependent on IP_3_R/Ca^2+^ signaling. Finally, we demonstrate that, in MDA-MB-231, a Bcl-xL-dependent breast cancer cell model, endogenous Bcl-xL can exploit this mechanism to suppress IP_3_R activity and to counteract Ca^2+^-driven apoptosis. Indeed, lowering Bcl-xL-protein levels in MDA-MB-231 cells resulted in augmented ATP-induced IP_3_R-mediated Ca^2+^ release and in increased sensitivity to staurosporine (STS). Overall, our data challenge the current paradigm that Bcl-xL promotes cell survival by sensitizing IP_3_Rs to IP_3_. Instead, we demonstrate that Bcl-xL inhibits IP_3_R function through a conserved lysine residue in its BH3 domain, thereby protecting cells against IP_3_R/Ca^2+^-driven apoptosis.

## Results

### Bcl-xL inhibits IP_3_R-mediated Ca^2+^ release in living cells

**Fig. 1:**
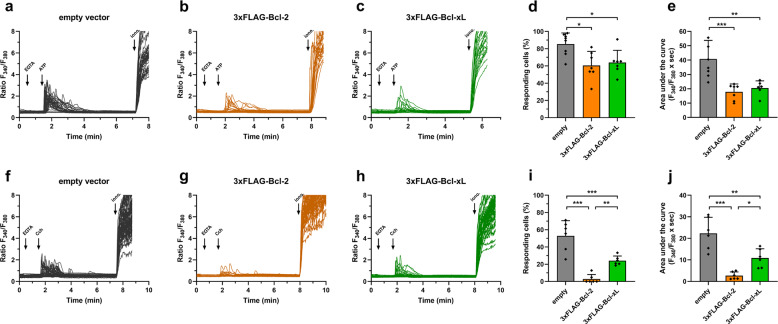
Bcl-xL overexpression inhibits IP_3_R-mediated Ca^2+^ release in living cells. Ca^2+^ signals were measured in Fura-2-loaded HEK cells expressing empty vector (pCMV24-P2A-mCherry; black), Bcl-2 (pCMV24-3xFLAG-Bcl-2-P2A-mCherry; orange) or Bcl-xL (pCMV24-3xFLAG-Bcl-xL-P2A-mCherry; green). EGTA (3 mM) was added to chelate extracellular Ca^2+^. IP_3_R-mediated Ca^2+^ release was evoked by ATP (10 µM) (**a**–**e**) or carbachol (Cch, 10 µM) (**f**–**j**). Ionomycin (iono, 5 µM diluted in 250 mM CaCl_2_) was added to assess the maximal Ca^2+^ response. Representative single-cell Ca^2+^ responses obtained from one well containing about 20–40 cells are shown (**a**–**c**, **f**–**h**). For each condition, six to nine different wells obtained from two to three independent transfections were monitored. Percentages of responding cells **(d**, **i**) and areas under the curve (**e**, **j**) were calculated from the Ca^2+^ traces. Data are represented as mean of wells ± SD (*N* = 6–9), each data point represents one well. Statistically significant differences were determined using a one-way ANOVA (*P < 0.05).

Bcl-xL has been reported to sensitize IP_3_Rs in living cells [26]. Here, we evaluated the effect of Bcl-xL overexpression on IP_3_R function by monitoring agonist-induced Ca^2+^ release (Fig. 1, Fig. S1). First, we performed Ca^2+^ measurements in a population-based assay, using the ratiometric fluorescent Ca^2+^ probe Fura-2 (Fig. S1). We used trypsin, an efficient agonist of protease activated receptors 2 in HEK-293 cells [29], thereby triggering IP_3_ formation. We elicited IP_3_R-mediated Ca^2+^ release in Fura-2-loaded HEK-3KO cells with reconstituted rIP_3_R1 (HEK-rIP_3_R1) and we studied the impact of overexpressing Bcl-xL. For this, we transfected the cells with either P2A-mCherry or 3xFLAG-Bcl-xL-P2A-mCherry. The 3xFLAG-Bcl-xL-P2A-mCherry construct generates separate mCherry and 3xFLAG-Bcl-xL proteins due to its P2A self-cleaving sequence. In contrast to previous reports [25, 26], Bcl-xL overexpression significantly reduced the amplitude (Fig. S1a, c) and the area under the curve of the Ca^2+^ signals (Fig. S1b, d) induced by both low (0.1 µM) and high (1 µM) trypsin concentrations. Moreover, similarly to our findings related to Bcl-2 and IP_3_R function [24], we noticed that the inhibitory effect of Bcl-xL overexpression was more prominent at low agonist concentrations than at high agonist concentrations.

This unexpected IP_3_R inhibition by Bcl-xL in population-based Ca^2+^ measurements prompted us to validate the effect of Bcl-xL overexpression on Ca^2+^ signals in single cells exposed to other agonists (Fig. 1) and to compare it with Bcl-2 overexpression, an established inhibitory modulator of IP_3_Rs [20, 22]. Single cell Ca^2+^ imaging was performed in Fura-2-loaded HEK-293 cells transfected with either P2A-mCherry, 3xFLAG-Bcl-xL-P2A-mCherry or 3xFLAG-Bcl-2-P2A-mCherry, whereby only mCherry-positive cells were analyzed. Here, we used ATP (10 µM) to trigger IP_3_R-mediated Ca^2+^ release (Fig. 1a, b, c). Similarly, we observed that Bcl-xL overexpression reduced the percentage of responding cells (Fig. 1d) and the area under the curve as representative of the extent of Ca^2+^ release (Fig. 1e). Interestingly, Bcl-xL appeared to dampen IP_3_R-mediated Ca^2+^ release to a similar extent as Bcl-2. We also measured the effect of Bcl-xL/Bcl-2 overexpression on Ca^2+^ signals elicited by another agonist, namely carbachol (Fig. 1f, g, h). We found that similarly to Bcl-2, Bcl-xL also inhibited carbachol-induced Ca^2+^ signals, although Bcl-xL appeared less potent than Bcl-2 (Fig. 1i, j). These results indicate that, similarly to Bcl-2, Bcl-xL inhibits IP_3_R-mediated Ca^2+^ release, irrespective of the extracellular agonist that is applied.

### Full-length Bcl-xL, but not its BH4 domain, targets the LBD of IP_3_R1

**Fig. 2:**
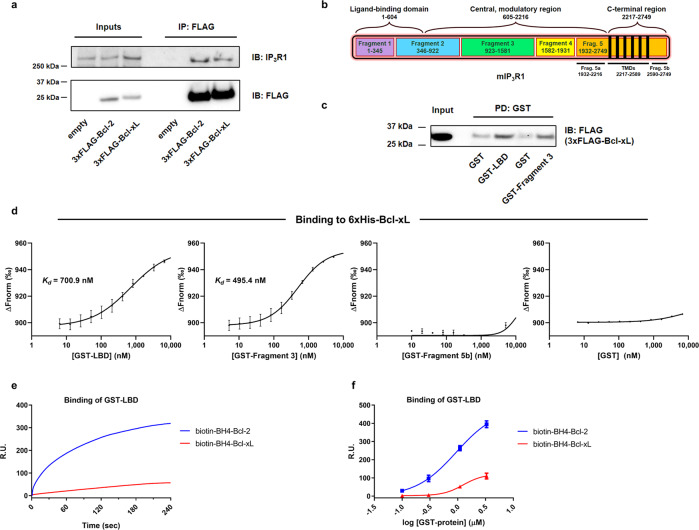
Bcl-xL, but not its BH4 domain, binds to IP_3_R1 involving LBD and Fragment 3. **a** Representative co-immunoprecipitation experiments using anti-FLAG performed in lysates from HeLa cells transiently overexpressing 3xFLAG-Bcl-2 or 3xFLAG-Bcl-xL. This experiment was performed three times using each time independently transfected and freshly prepared cell lysates. The samples were analyzed via western blot using antibodies against IP_3_R1 and FLAG. Total HeLa lysates were used as input (20 µg). PD: pull down; IB: immunoblot. **b** Linear representation of a mouse IP_3_R1 (mIP_3_R1) monomer. The three functional domains, including the ligand-binding domain (LBD), and the five tryptic fragments, including Fragment 3, are represented. Respective amino acids are indicated by numbers. TMDs: transmembrane domains. **c** Representative GST-pull down experiment for assessing the binding of 3xFLAG-Bcl-xL from COS-7 cell lysate to GST-fused IP_3_R1 fragments. The samples were analyzed via western blot. Total COS-7 lysate was used as input (0.1 µg). This experiment was performed four times utilizing each time independently transfected and freshly prepared cell lysates. PD: pull down; IB: immunoblot. The corresponding western blot for the GST-IP_3_R fragments was shown in Fig. S2a. **d** Binding curves showing the interaction of purified 6xHis-Bcl-xL with titrated GST-fused IP_3_R domains generated by MST. GST was used as a negative control. Concentration of the 6xHis-Bcl-xL was kept constant at 50 nM, whereas the GST-LBD, GST-Fragment 3, GST-Fragment 5b and parental GST proteins were titrated down from 15 µM to 5 nM. The unit of the left axis (Δ*F*_norm_) is a ratio of normalized fluorescence. Data points represent mean ± SD from triplicate measurements. **e** Representative sensorgrams of SPR experiments showing the binding properties of GST-fused IP_3_R-LBD, applied at 1.1 µM, to biotin-BH4-Bcl-xL and biotin-BH4-Bcl-2 peptides. The biotin-BH4 peptides, immobilized on a streptavidin-coated sensor chip. Sensorgrams were obtained after background correction for binding to the scrambled peptides. Data are expressed in resonance units (R.U.) as a function of time. **f** Quantitative analysis of the binding properties of biotin-BH4-Bcl-2 and biotin-BH4-Bcl-xL peptides to GST-LBD measured by SPR. Values obtained from independent experiments were plotted as mean ± SEM (*N* = 4).

Next, we elucidated the interaction between Bcl-xL and IP_3_R. First, we compared the interaction of Bcl-xL and Bcl-2 with full-length IP_3_R. We overexpressed 3xFLAG-tagged Bcl-xL or Bcl-2 in HeLa cells expressing endogenous IP_3_Rs, immunoprecipitated Bcl-xL or Bcl-2 using anti-FLAG-coupled beads and immunoblotted for IP_3_Rs (Fig. 2a). This co-immunoprecipitation (coIP) analysis revealed that Bcl-xL could immunoprecipitate IP_3_Rs to a rather similar extend as Bcl-2, indicating that Bcl-xL and Bcl-2 display quite similar IP_3_R-binding properties. Bcl-2 can bind to the central, modulatory region of IP_3_R1, specifically to tryptic Fragment 3 [8, 12, 27]. We demonstrated that Bcl-2 binding to this site is associated with inhibition of IP_3_R1 activity. In addition to this, we recently discovered that Bcl-2 could also bind to the LBD of IP_3_R1, indicating that multiple regions are involved in IP_3_R1/Bcl-2 complex formation and inhibition of channel activity [11]. We also found that Bcl-xL could target this central, modulatory region of IP_3_R1 though with lower efficiency than Bcl-2 [12]. However, given the prominent inhibition of IP_3_Rs by Bcl-xL and the observation that this inhibition appears dependent on the agonist concentration, we asked whether Bcl-xL could also target the LBD. Thus, we used lysates from COS-7 cells that overexpressed 3xFLAG-Bcl-xL in GST-pull down experiments against purified GST-LBD and GST-Fragment 3 (representing a major part of the central, modulatory region) of IP_3_R1 (Fig. 2b). Our analysis revealed that similarly to Bcl-2, Bcl-xL can bind to both regions (Fig. 2c, Fig. S2a). Since GST-pulldowns are only semi-quantitative, we applied microscale thermophoresis (MST), a biophysical approach allowing to measure molecular interactions. This technique is based on detecting a change in fluorescence of a labeled target as a function of the concentration of a non-fluorescent ligand. The change in fluorescence reflects the thermophoretic movement of the fluorescent target subjected to a microscopic temperature gradient. We thus used MST to assess direct binding between purified GST-IP_3_R fragments and purified 6xHis-Bcl-xL to determine the binding affinity. Using MST, we demonstrated that both purified GST-LBD and GST-Fragment 3 could bind to wild-type 6xHis-Bcl-xL (Fig. 2d). The specificity of this interaction is underpinned by two negative controls, parental GST and GST-Fragment 5b that lacks the 6^th^ TMD, previously established to be critical for Bcl-xL binding [12]. Indeed, no binding between 6xHis-Bcl-xL and GST or GST-Fragment 5b could be detected. Furthermore, we obtained the dissociation constant for both domains with 6xHis-Bcl-xL, revealing a *K*_*d*_ of ~701 nM for 6xHis-Bcl-xL interaction with the GST-LBD and a *K*_*d*_ of ~495 nM for 6xHis-Bcl-xL interaction with GST-Fragment 3. This indicates that wild-type 6xHis-Bcl-xL binds to both the LBD and Fragment 3.

We previously characterized the binding characteristics of the BH4 domain of Bcl-2 and Bcl-xL with Fragment 3 in detail via surface plasmon resonance (SPR) [27]. This study showed that the BH4 domain of Bcl-2 but not the one of Bcl-xL could interact with the Fragment 3. Recently, we also identified a novel binding site for Bcl-2’s BH4 domain in the LBD, but had not yet characterized its ability to interact with Bcl-xL’s BH4 domain [24]. Thus, we examined the importance of the BH4 domain of Bcl-xL for binding to the LBD using SPR. Biotin coupled to a peptide encompassing BH4-Bcl-xL was immobilized on streptavidin chips and different concentrations of purified LBD were applied as an analyte. Background binding was determined using a peptide with a scrambled sequence and subtracted. Biotin-BH4-Bcl-2 was used as a positive control for detecting LBD binding. The association curves for 1.1 µM GST-LBD show prominent binding to BH4-Bcl-2 while its binding to BH4-Bcl-xL is much lower (Fig. 2e). Similarly to what was observed for the binding of Fragment 3 to BH4-Bcl-2 *versus* BH4-Bcl-xL [27], GST-LBD displayed a strong concentration-dependent binding to immobilized BH4-Bcl-2 [11], while its binding to immobilized BH4-Bcl-xL appeared much weaker (Fig. 2f).

Taken together, these data reveal that while both Bcl-2 and Bcl-xL target the same regions in IP_3_R, they employ different binding determinants for these interactions. In contrast to Bcl-2, which exploits its BH4 domain for binding to LBD [24] and Fragment 3 [27], Bcl-xL seems to interact with the same IP_3_R regions but via motifs located outside of the BH4 domain.

### Residue K87 of Bcl-xL contributes to the interaction with IP_3_R and particularly to the binding to LBD and Fragment 3

**Fig. 3:**
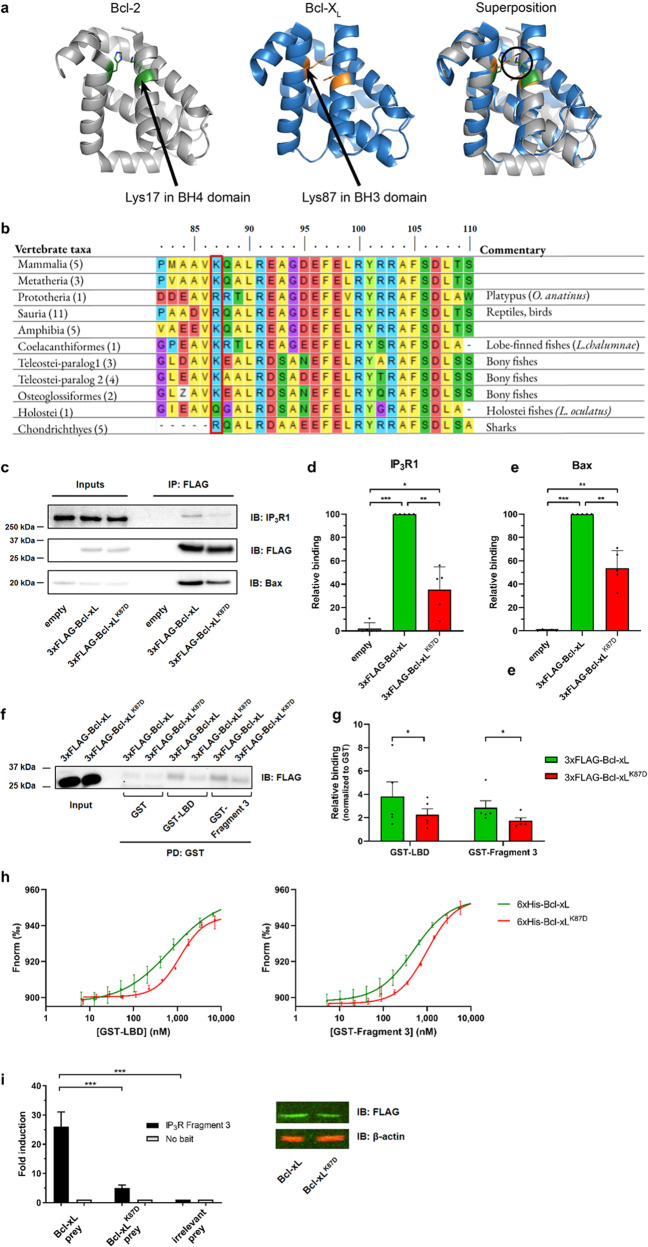
The K87 residue is critical for Bcl-xL interaction with IP_3_R. **a** *In silico* representations of Bcl-2 and Bcl-xL three-dimensional structures. The lysine residues of interest (K17 in Bcl-2 and K87 in Bcl-xL) are indicated. Image taken from our previously published work [28]; this work is licensed under a Creative Commons Attribution 4. 0 International License. **b** Alignment of the conserved amino acid motifs for Bcl-xL’s BH3 domain in vertebrates. The conserved lysine (K87 in human) is highlighted (red rectangle). The number of species used for each motif construction is shown in parentheses. “Z” means glutamic acid or glutamine. Top numbers represent amino acid numbers in human Bcl-xL sequence. **c** Representative co-immunoprecipitation (coIP) experiments using anti-FLAG performed in lysates from HeLa cells transiently overexpressing 3xFLAG-Bcl-xL or 3xFLAG-Bcl-xL^K87D^. The samples were analyzed via western blot using antibodies against IP_3_R1, FLAG and Bax. Total HeLa lysates were used as input (10 µg). PD: pull down; IB: immunoblot. The immunoreactive bands from independent coIP experiments, using each time independently transfected cells and freshly prepared lysates, were quantified and normalized to the binding of IP_3_R1 (**d**) and Bax (**e**) to 3xFLAG-Bcl-xL. Data represent mean ± SD (*N* = 5). Statistically significant differences were determined using a one-way ANOVA (*P < 0.05). **f** Representative GST-pull down experiment comparing the binding of 3xFLAG-Bcl-xL *vs*. 3xFLAG-Bcl-xL^K87D^ from COS-7 cell lysate to purified GST-fused IP_3_R1 fragments and parental GST control. The samples were analyzed via western blot using anti-FLAG. Total COS-7 lysates were used as input (0.1 µg). The corresponding western blot for the GST-IP_3_R fragments is shown in Fig. S2b. **g** The immunoreactive bands from independent GST-pull down experiments, using each time independently transfected cells and freshly prepared lysates, were quantified and normalized to the binding of 3xFLAG-Bcl-xL and 3xFLAG-Bcl-xL^K87D^ to parental GST control, which was set as 1 for each experiment. The data are plotted as mean ± SD (*N* = 5). Statistically significant differences were determined using paired *t* test (*P < 0.05). **h** Binding curves showing the interaction of purified 6xHis-Bcl-xL and 6xHis-Bcl-xL^K87D^ with titrated GST-fused IP_3_R domains generated by MST. Concentration of the 6xHis-Bcl-xL and 6xHis-Bcl-xL^K87D^ targets was kept constant at 50 nM, whereas the GST-LBD and GST-Fragment 3 were titrated down from 15 µM to 5 nM. The unit of the left axis (ΔF_norm_) is a ratio of normalized fluorescence. The binding curves of wild-type 6xHis-Bcl-xL with GST-fused proteins are represented from Fig. 2d and are shown as reference. The binding curve of 6xHis-Bcl-xL^K87D^ with parental GST is shown in Fig. S4. Data points represent mean ± SD from triplicate measurements. **i** Left: Representative example of a MAPPIT experiment. The binding is shown as fold induction, calculated by dividing the average luciferase activity of erythropoietin-stimulated cells by the average obtained in non-stimulated cells. Binding of Bcl-xL, the Bcl-xL^K87D^ mutant or irrelevant prey control (SV40 large T antigen) to the IP_3_R Fragment 3 and as negative control the bait vector without Fragment 3 are shown. Fold induction values at least four times higher than the irrelevant prey control are considered as bona fide protein-protein interactions. Values are represented as the mean of triplicates ± SEM within one representative experiment. The experiment was independently performed four times (*N* = 4). Statistically significant differences were determined using a one-way ANOVA (*P < 0.05). Right: Odyssey western blot analyses for the FLAG tag of the prey vector containing Bcl-xL or the Bcl-xL^K87D^ mutant fusion proteins (green) or for β-actin (red).

Our previously published results [27] and the data reported here (Fig. 2f) indicate that, in contrast to the BH4-Bcl-2, the BH4-Bcl-xL could not be responsible for targeting LBD and Fragment 3 of IP_3_R1. We therefore aimed to elucidate the molecular determinants in Bcl-xL responsible for its interaction with IP_3_Rs. Since Bcl-xL targets the same IP_3_R regions as Bcl-2, we envisioned that a similar interaction surface could underlie this phenomenon. We previously showed that K17 located in the middle of the BH4-Bcl-2 was critical for binding and inhibiting IP_3_Rs [27]. In the BH4-Bcl-xL, the corresponding residue is not a lysine but an aspartate, preventing its ability to bind to IP_3_Rs. However, a previously performed *in silico* Bcl-2/Bcl-xL structure superposition revealed that K87, located in the BH3 domain of Bcl-xL (BH3-Bcl-xL), likely is spatially constrained in a similar manner to K17 of BH4-Bcl-2 (Fig. 3a) [28]. Moreover, sequence analysis of Bcl-xL orthologs among main vertebrate lineages revealed that K87 is highly conserved (Fig. 3b) and thus the importance of this residue was examined further. Interestingly, this lysine is located on the opposite side of the binding pocket involved in the interaction with Bak and Bax.

First, we used confocal microscopy to assess whether altering K87 into an aspartate affected Bcl-xL’s subcellular localization. We transfected HeLa cells to express a mitochondria- or an ER- targeted RFP and compared the localization of 3xFLAG-Bcl-xL *versus* 3xFLAG-Bcl-xL^K87D^ using anti-FLAG-based immunofluorescence (Fig. S3a–d). We calculated average Pearson’s coefficients above 0.75 for all conditions (Fig. S3e, f), indicating a high colocalization of Bcl-xL and Bcl-xL^K87D^ with both the mitochondria and the ER. We also calculated average Manders’ M1 coefficient to quantify the fraction of Bcl-xL or Bcl-xL^K87D^ overlapping with the mitochondria, being 0.8 for both Bcl-xL and Bcl-xL^K87D^, or with the ER, being 0.6 for both Bcl-xL and Bcl-xL^K87D^ (Fig. S3g, h). The similar coefficients calculated for the wild-type Bcl-xL and the Bcl-xL^K87D^ indicate that the K87D mutation in Bcl-xL did not alter its subcellular localization.

Second, we tested the effect of the K87D mutation on the interaction of Bcl-xL with full-length IP_3_R. We therefore overexpressed 3xFLAG-tagged Bcl-xL or Bcl-xL^K87D^ in HeLa cells expressing endogenous IP_3_Rs (Fig. 3c), immunoprecipitated Bcl-xL or Bcl-xL^K87D^ using anti-FLAG-coupled beads and immunoblotted for IP_3_Rs. This co-immunoprecipitation analysis revealed that Bcl-xL^K87D^ binding to the IP_3_R channel is severely impaired compared to wild-type Bcl-xL (Fig. 3c, d). We also immunoblotted for Bax to determine Bax binding to Bcl-xL or Bcl-xL^K87D^. We found that both Bcl-xL and Bcl-xL^K87D^ could bind Bax, though Bax binding to Bcl-xL^K87D^ appeared slightly reduced compared to its binding to Bcl-xL (Fig. 3c, e).

Third, we performed GST-pull down experiments with lysates from COS-7 cells overexpressing 3xFLAG-Bcl-xL or 3xFLAG-Bcl-xL^K87D^ (Fig. 3f, Fig. S2b). We compared their binding to purified GST-LBD and GST-Fragment 3 of IP_3_R1. In comparison to wild-type Bcl-xL, the ability of Bcl-xL^K87D^ to bind the LBD and the Fragment 3 appears significantly reduced (Fig. 3f, g).

Fourth, we used MST to quantitatively assess the interaction of purified GST-LBD and GST-Fragment 3 with purified 6xHis-Bcl-xL^K87D^, similarly to the experiment performed using wild-type 6xHis-Bcl-xL (Fig. 2d). We showed that although 6xHis-Bcl-xL^K87D^ could interact with both IP_3_R domains, it was with lower affinity than wild-type 6xHis-Bcl-xL (Fig. 3h). Of note, 6xHis-Bcl-xL^K87D^ did not interact with GST (Fig. S4). Indeed, 6xHis-Bcl-xL^K87D^ displayed higher dissociation constants than wild-type 6xHis-Bcl-xL for the interaction with the IP_3_R fragments. GST-LBD: *K*_*d*_ ~1166 nM (with 6xHis-Bcl-xL^K87D^) *versus K*_*d*_ ~701 nM (with 6xHis-Bcl-xL). GST-Fragment 3: *K*_*d*_ ~990 nM (with 6xHis-Bcl-xL^K87D^) *versus K*_*d*_ ~495 nM (with 6xHis-Bcl-xL). Thus, the GST-pull down assays and MST analysis indicate that, compared to wild-type Bcl-xL, Bcl-xL^K87D^ is impaired in binding to LBD and Fragment 3.

Finally, we applied an *in cellulo* mammalian protein–protein interaction trap (MAPPIT) assay, which is based on the functional complementation of cytokine receptor signaling [30]. The MAPPIT data confirmed that Bcl-xL is able to interact with Fragment 3 and that the interaction was impaired by the introduction of the K87D mutation (Fig. 3i). No binding was detected with the negative control, indicating that the interaction is specific. In this assay, Bcl-xL binding to LBD could not be observed, potentially due to interference of the fusion protein to establish a functional recomplementation of the cytokine receptor.

Taken together, our data demonstrate that the K87 residue is crucial for the interaction of Bcl-xL with the IP_3_R, where it is involved in its binding to both the LBD and the Fragment 3.

### K87 residue is critical for Bcl-xL-mediated IP_3_R inhibition in living cells

**Fig. 4:**
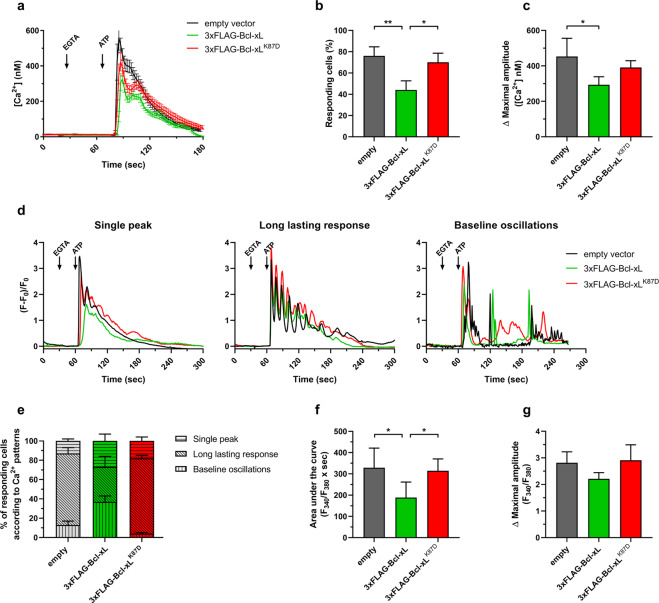
Bcl-xL, but not Bcl-xL^K87D^, overexpression inhibits IP_3_R-mediated Ca^2+^ release in single cells. Calcium measurements obtained from Fura-2-loaded HeLa (**a–c**) and COS-7 cells (**d–g**) transfected with a Bcl-xL (pCMV24-3xFLAG-Bcl-xL-P2A-mCherry; green) or Bcl-xL^K87D^-coding vector (pCMV24-3xFLAG-Bcl-xL^K87D^-P2A-mCherry; red), or an empty vector (pCMV24-P2A-mCherry; black). ER Ca^2+^ response is elicited by addition of 70 nM (HeLa) or 500 nM (Cos-7) ATP, after addition of 3 mM EGTA to chelate extracellular Ca^2+^. Ionomycin (5 µM) diluted in 250 mM CaCl_2_ was added at the end of the experiment (not shown) to trigger a high Ca^2+^ release and confirm all the cells are equally loaded with Fura-2. For each condition, three to five wells were monitored and about 20–30 cells were analyzed by well. **a** Representative traces of Ca^2+^ release in COS-7 cells. Traces represent mean ± SEM of one representative measurement (one well, about 20–30 cells). Percentage of responding cells (**b**) and maximal peak amplitude (**c**) were calculated for each condition. Data represent mean ± SD of four independent experiments (*N* = 4). Statistically significant differences were determined using a one-way ANOVA (*P < 0.05). Non-responding cells are defined as cells in which fluorescence signal measured after ATP stimulation do not exceed the maximal fluorescence value + SEM measured before stimulation. **d** Representative traces of Ca^2+^ release in HeLa cells. Traces represent diverse Ca^2+^ release patterns for one single cell. Distribution of typical Ca^2+^ release patterns (**e**), areas under the curve (**f**) and amplitudes of the maximal Ca^2+^ peak (**g**) were calculated from the Ca^2+^ traces of the responding cells. Data represent mean ± SD of four independent experiments (*N* = 4). Statistically significant differences were determined using a one-way ANOVA (*P < 0.05).

Next, we examined the role of the K87 residue in Bcl-xL-mediated IP_3_R inhibition. We used Fura-2-loaded COS-7 (Fig. 4a–c) and HeLa (Fig. 4d–g) cells with overexpressed mCherry and either Bcl-xL or Bcl-xL^K87D^. We used mCherry to identify transfected cells. We first studied the effect of Bcl-xL and Bcl-xL^K87D^ overexpression in COS cells on IP_3_R-mediated Ca^2+^ signals elicited by 500 nM ATP, a relatively high concentration provoking a Ca^2+^ response in about 75% of the cells. Extracellular Ca^2+^ was chelated with EGTA, so the reported Ca^2+^ signals only originate from internal stores. Under these conditions, ATP-induced Ca^2+^ signals appeared as a single transient (Fig. 4a). While about 75% of the cells expressing the empty vector displayed a response to ATP, only 40% of the cells expressing Bcl-xL responded (Fig. 4b). Cells expressing Bcl-xL^K87D^ displayed similar responsiveness to ATP as empty vector-expressing cells with about 75% responding cells. Quantification of the amplitude of the ATP-induced Ca^2+^ transient in the responding cells yielded similar trends (Fig. 4c). Overexpression of Bcl-xL provoked a decrease in the peak [Ca^2+^] provoked by ATP, while overexpression of Bcl-xL^K87D^ failed to do this.

Then, we aimed to study the effect of Bcl-xL in HeLa cells, well-known to display long-lasting Ca^2+^ oscillations in response to extracellular agonists [31, 32]. Here, we exposed HeLa cells to a very low [ATP] (70 nM), thereby mimicking basal, pro-survival Ca^2+^ oscillations and enhancing the likelihood of observing different Ca^2+^-signaling patterns. We could discriminate three distinct Ca^2+^-signaling profiles: single peak responses, long-lasting responses and baseline Ca^2+^ oscillations (Fig. 4d). Consistent with an inhibitory effect of Bcl-xL on IP_3_Rs, we found that long-lasting responses were clearly impaired upon overexpression of Bcl-xL (Fig. 4d, e). Interestingly, this effect was not observed upon overexpression of Bcl-xL^K87D^. Furthermore, the area under the curve (Fig. 4f) and the peak amplitude (Fig. 4g) were reduced upon overexpression of Bcl-xL, but not Bcl-xL^K87D^. This demonstrates that Bcl-xL’s inhibitory action on IP_3_Rs is critically dependent on the K87 residue.

Finally, we also determined that overexpressed Bcl-xL or its mutant did not alter the ER Ca^2+^ store content, by monitoring ER Ca^2+^ release in HeLa cells following sarco/endoplasmic reticulum Ca^2+^ ATPase (SERCA) inhibition by 1 µM thapsigargin (Fig. S5). These data are consistent with the differences observed upon ATP stimulation not being as a result of altered ER Ca^2+^ levels, but instead are due to the specific effect of Bcl-xL on IP_3_R-mediated Ca^2+^ release.

### Purified Bcl-xL can directly suppress IP_3_R single-channel opening

As all our functional data were obtained in intact cells, we also wished to provide more direct evidence for IP_3_R inhibition by Bcl-xL through electrophysiology. This is important because in intact cell systems Bcl-xL may have other targets besides IP_3_Rs that impact cytosolic Ca^2+^ signals. In addition, these experiments can be performed in tightly controlled conditions, including different IP_3_ and Bcl-xL concentrations. Therefore, we aimed to study the impact of purified Bcl-xL proteins on IP_3_Rs. We generated 6xHis-tagged versions of full-length Bcl-xL, Bcl-xL^ΔTMD^ and full-length Bcl-xL^K87D^ that enabled their purification from *E. coli* using NiNTA columns (Fig. S6a).

**Fig. 5:**
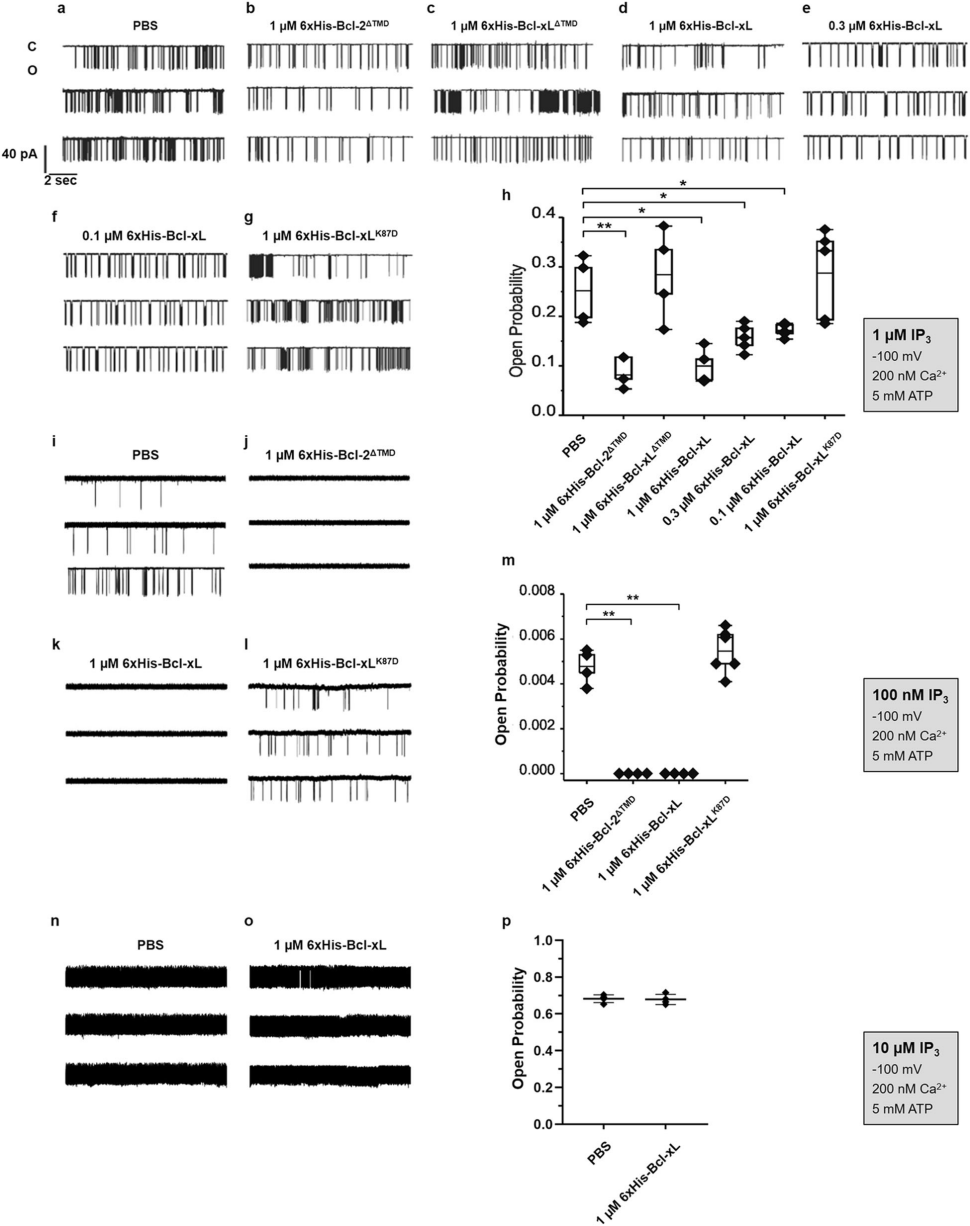
Purified Bcl-xL, but not Bcl-xL^K87D^, suppresses IP_3_R1 single-channel opening. Representative IP_3_R1 single-channel recordings from DT40-3KO cells ectopically expressing IP_3_R1. The channel opening was evoked by 1 µM IP_3_ (**a**–**h**), 100 nM IP_3_ (**i** to **m**) or 10 µM IP_3_ (**n**–**p**) at 200 nM Ca^2+^ and 5 mM ATP in the presence of PBS (**a**, **i**, **n**) or in the presence of 1 μM 6xHis-Bcl-2^ΔTMD^ (**b**, **j**), 1 μM 6xHis-Bcl-xL^ΔTMD^ (**c**), 1 μM 6xHis-Bcl-xL (**d**, **k**, **o**), 0.3 μM 6xHis-Bcl-xL (**e**), 0.1 μM 6xHis-Bcl-xL (**f**), or 1 μM 6xHis-Bcl-xL^K87D^ (**g**, **l**) purified proteins. Each vertical bar represents one single-channel current recording; C: closed; O: opened. Histogram depicting the *P*_o_ ± SEM (*N* = 4 or 5) for the IP_3_R1 current recordings (**h**, **m**, **p**). Statistically significant differences were determined using a one-way ANOVA (*P < 0.05).

Next, we tested the effect of the different recombinantly expressed and purified 6xHis-Bcl-xL variants on IP_3_R1 single-channel activity using the on-nucleus patch-clamp technique (Fig. 5). Channel opening in isolated nuclei obtained from DT40-3KO cells ectopically expressing IP_3_R1 was triggered by 1 μM of IP_3_ (Fig. 5a). We used purified Bcl-2^ΔTMD^ as a benchmark (Fig. 5b), which we previously validated to inhibit IP_3_R1 single-channel openings [24]. We subsequently first tested whether Bcl-xL^ΔTMD^ could inhibit the opening of IP_3_R1 channels induced by 1 µM IP_3_, but this protein failed to modulate (inhibit/sensitize) IP_3_R1 channels (Fig. 5c). Next, we assessed the effect of 1 µM full-length Bcl-xL (Fig. 5d), a concentration previously proposed to have a stimulatory effect on IP_3_R [26]. Consistent with the data obtained in intact cells and similarly to 1 µM Bcl-2^ΔTMD^ (Fig. 5b), application of 1 μM full-length 6xHis-Bcl-xL resulted in a significantly decreased open probability (*P*_*O*_) of IP_3_R1 channels in the presence of 1 µM IP_3_ (Fig. 5d). Clearly, these results contradict previous data that reported that Bcl-xL sensitizes IP_3_R [25, 26]. In these reports, the effect of Bcl-xL on IP_3_Rs was shown to exhibit a bell-shaped dependence with 1 µM of Bcl-xL optimally sensitizing IP_3_Rs [26]. Hence, to ensure that we did not apply Bcl-xL at too high concentrations, we also examined the effects of 300 nM (Fig. 5e) and 100 nM (Fig. 5f) full-length Bcl-xL. These lower Bcl-xL concentrations also inhibited IP_3_R1 single-channel opening, though with lower potency compared to 1 µM full-length Bcl-xL. Next, we examined the effect of 1 µM full-length Bcl-xL^K87D^ protein on IP_3_R1 single-channel openings activated by 1 µM IP_3_ (Fig. 5g). Consistent with our in vitro binding assays and our Ca^2+^-imaging studies in intact cells, Bcl-xL^K87D^ failed to inhibit IP_3_R1 single-channel activity. Quantification of all conditions is shown in Fig. 5h. These data further demonstrate that Bcl-xL has a direct inhibitory effect on IP_3_R activity and that K87 is critical for this effect.

Another potential explanation could be that the conditions in which we have measured IP_3_R1 opening favor the detection of inhibitory effects and we may have missed potential sensitizing effects. We therefore measured the impact of purified Bcl-xL proteins on IP_3_R1 single-channel openings induced by threshold concentrations of IP_3_ (Fig. 5i–m). In the presence of 100 nM IP_3_, the *P*_*O*_ was reduced to ~0.005 (Fig. 5i), compared to a *P*_*O*_ of ~0.25 at 1 µM IP_3_ (Fig. 5a). Such conditions, which initiate threshold IP_3_R1 opening, should favor the detection of any potential sensitization of the channel. Nevertheless, similarly to Bcl-2^ΔTMD^ (Fig. 5j), full-length Bcl-xL provoked a complete inhibition IP_3_R1 opening (Fig. 5k), while Bcl-xL^K87D^ failed to inhibit IP_3_R1 opening (Fig. 5l). Quantification of all conditions is shown in Fig. 5m.

Finally, we also performed electrophysiology experiments to assess IP_3_R inhibition by 6xHis-Bcl-xL with high IP_3_ concentrations (Fig. 5n–p). Indeed, we previously demonstrated that high IP_3_ concentrations could compete with Bcl-2 for binding to the LBD of IP_3_Rs, thereby alleviating IP_3_R inhibition by Bcl-2 [24]. Here, we have established that 6xHis-Bcl-xL is also able to interact with the LBD of IP_3_Rs, prompting us to test the effect of purified 6xHis-Bcl-xL on IP_3_R1 single-channel activity triggered by high concentration of IP_3_. With 10 µM IP_3_, the *P*_*O*_ reached more than 0.65 (Fig. 5n), compared to a *P*_*O*_ of about 0.25 at 1 µM IP_3_ (Fig. 5a). In those conditions, 6xHis-Bcl-xL did not alter channel activity, reflecting a loss of capacity to inhibit the channel at high [IP_3_] (Fig. 5o, p). These results suggest that, similarly to our observations made for Bcl-2, IP_3_ might compete with Bcl-xL for the LBD of IP_3_Rs, thereby rendering Bcl-xL less effective in inhibiting IP_3_Rs at high IP_3_ concentrations.

**Fig. 6:**
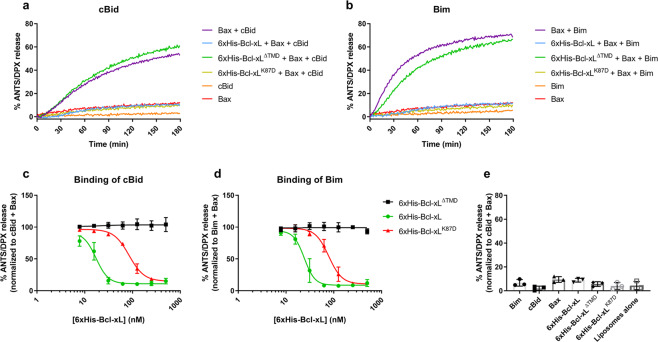
Purified Bcl-xL and Bcl-xL^K87D^ proteins inhibit Bax-mediated permeabilization of liposomes. ANTS/DPX-encapsulated liposomes were incubated with 100 nM unlabeled Bax and 20 nM unlabeled cBid (**a** and **c**) or Bim (**b** and **d**), as well as a range of seven concentrations (from 500 to 8 nM) of unlabeled 6xHis-Bcl-xL, 6xHis-Bcl-xL^K87D^ or 6xHis-Bcl-xL^ΔTMD^ purified proteins. Upon Bax oligomerization and -pore formation, fluorescence increases due to loss of fluorescent molecule-quencher proximity. **a** and **b** Representative traces of liposomes permeabilization experiments using 500 nM of purified 6xHis-Bcl-xL proteins. Liposomes permeabilization is represented as the percentage of ANTS/DPX release. **c** and **d** Summary of liposomes permeabilization experiments performed with the concentration range of 6xHis-Bcl-xL proteins mentioned before. ANTS/DPX release was normalized to the maximal permeabilization (Bax + cBid or Bim, in absence of Bcl-xL proteins). Each point represents mean ± SEM (*N* = 3). **e** ANTS/DPX release was measured in the presence of single purified proteins (20 nM Bim, 20 nM cBid, 100 nM Bax, or 500 nM Bcl-xL variants) or with liposomes only. Data represents means ± SEM (*N* = 3).

Given that our findings were diametrically opposite to the previously reported findings, we sought to validate that the purified full-length 6xHis-Bcl-xL proteins used were properly folded and displayed bona fide anti-apoptotic functions. We first determined the CD spectrum of both Bcl-xL, which indicated that wild-type Bcl-xL, Bcl-xL^K87D^ and Bcl-xL^ΔTMD^ had a proper α-helical folding (Fig. S6b). Moreover, we performed a thermal ramping experiment. Unfolding of wild-type Bcl-xL was characterized by two apparent melting temperatures Tm1: 67 °C and Tm2: 55.47 °C, which were shifted to the left for Bcl-xL^K87D^ (Tm1: 46.11 °C and Tm2: 53.2 °C), indicating some destabilizing effect of the mutation. These observations very much resemble the effect of K17D mutation in purified Bcl-2 [27]. We also measured Bcl-xL^ΔTMD^, which was characterized by one melting temperature Tm1: 76 °C, indicating that Bcl-xL^ΔTMD^ is much more stable than wild-type Bcl-xL (Fig. S6c). Next, we employed an in vitro Bax-liposome permeabilization assay, where purified Bax is incubated with liposomes encapsulating both a quencher (DPX) and a fluorophore (ANTS) (Fig. 6). Bax-pore formation can be triggered by cBid (Fig. 6a) or Bim (Fig. 6b) proteins, two “activator” BH3-only proteins. Full-length 6xHis-Bcl-xL potently inhibited cBid and Bim-triggered Bax-pore formation (IC_50_ of about 20 nM). Of note, 6xHis-Bcl-xL^K87D^ too inhibited Bax-pore formation, but was less efficacious (IC_50_ of about 80 nM) than 6xHis-Bcl-xL (Fig. 6c, d). This might relate to the reduced Bax binding observed in the coIPs using cell lysates (Fig. 3c). Consistent with previous reports [33], Bcl-xL^ΔTMD^ failed to inhibit Bax-pore formation.

Overall, our electrophysiological studies provide strong evidence that recombinant Bcl-xL with validated anti-apoptotic properties directly inhibits IP_3_R1 single-channel opening with a critical role for K87 in Bcl-xL. Furthermore, the data suggest that Bcl-xL’s TMD is not only important for inhibiting Bax [33, 34] but also for inhibiting IP_3_R opening. Yet, the significance and the role of the TMD of Bcl-xL in a cellular context for IP_3_R modulation remains to be elucidated.

### Bcl-xL^K87D^ is impaired in protecting cells against staurosporine-induced apoptosis

**Fig. 7:**
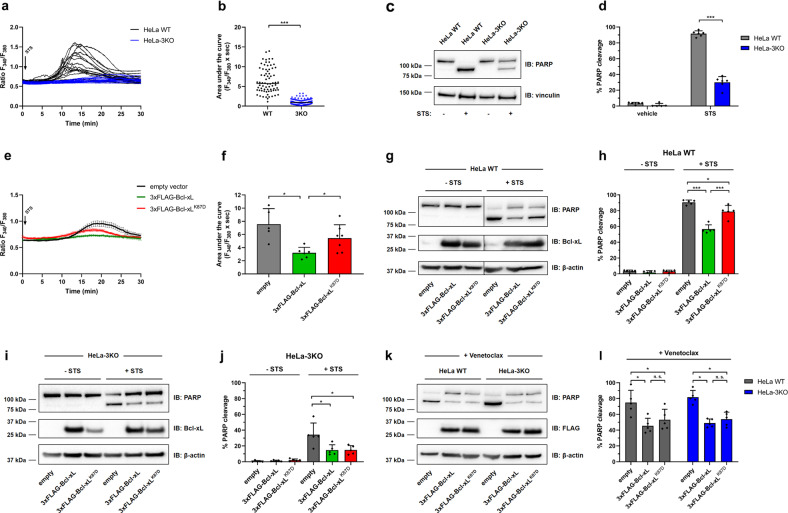
Wild-type Bcl-xL, but not Bcl-xL^K87D^, protects HeLa cells against IP_3_R/Ca^2+^-driven cell death using staurosporine. **a**, **b** Ca^2+^ measurements in Fura-2-loaded wild-type HeLa (black) and HeLa-3KO cells (blue). Cells were exposed to 0.5 µM staurosporine (STS), after addition of 3 mM EGTA to chelate extracellular Ca^2+^ (not shown). Representative traces of Ca^2+^ release are shown (**a**), along with areas under the curve (**b**) calculated for one hour following STS addition. For each experiment (*N* = 2), two wells were monitored per condition and about 20–30 cells were analyzed by well. Each trace and each point represent one cell. Statistically significant differences were determined using a *t* test (*P < 0.05). **c**, **d** Wild-type HeLa and HeLa-3KO cells were treated with 0.5 µM STS or vehicle (DMSO) for 6 h. The samples were analyzed via western blot (IB: immunoblot). Representative western blots assessing uncleaved (top band) and cleaved PARP (lower band) as well as vinculin (**c**). The immunoreactive bands from independent experiments, using each time freshly prepared cell lysates, were quantified (**d**) and PARP cleavage was calculated as the ratio of cleaved PARP over total PARP. The data are plotted as mean ± SD (*N* = 6). Statistically significant differences between the “+STS” conditions were determined using a *t* test (paired, two-tailed, *P < 0.05). **e**, **f** Ca^2+^ signals were measured in Fura-2-loaded wild type HeLa expressing empty vector (pCMV24-P2A-mCherry; black), Bcl-xL (pCMV24-3xFLAG-Bcl-xL-P2A-mCherry; green) or Bcl-xL^K87D^ (pCMV24-3xFLAG-Bcl-xL^K87D^-P2A-mCherry; red). ER Ca^2+^ release is triggered as in **a**. The traces represent the average response of all cells ± SEM in one well containing about 20 cells (**e**). The individual Ca^2+^ traces are shown in Fig. S7a. For each condition, 1 to 2 independent wells obtained from 4 different transfections were monitored. The areas under the curve were calculated for all individual cell during the 45 min following STS addition (**f**). Data are represented as mean of wells ± SD (*N* = 5 to 7), each data point represents one well. Statistically significant differences were determined using a one-way ANOVA (*P < 0.05). Another graphical representation is shown in Fig. S7b. **g**–**j** Wild type HeLa (**g**, **h**) and HeLa-3KO cells (**i**, **j**) transiently overexpressing 3xFLAG-Bcl-xL or 3xFLAG-Bcl-xL^K87D^ were treated with 0.5 µM STS or vehicle (DMSO) for 6 h. The samples were analyzed via western blot. Representative western blots assessing uncleaved and cleaved PARP as well as total Bcl-xL (endogenous + overexpressed) and β-actin (**g**, **i**). The vertical line in panel g indicates that two different parts of the same gel and exposure time were merged together. The original uncropped picture is shown in Fig. S7c. The immunoreactive bands from independent experiments, using each time independently transfected and treated cells and freshly prepared cell lysates, were quantified (**h**, **j**) and PARP cleavage was calculated like in **d**. The data are plotted as mean ± SD (*N* = 5). Statistically significant differences between the “+STS” conditions were determined using a one-way ANOVA (*P < 0.05). **k**, **l** Wild-type HeLa and HeLa-3KO cells transiently overexpressing 3xFLAG-Bcl-xL or 3xFLAG-Bcl-xL^K87D^ were treated with 25 µM venetoclax or vehicle (DMSO) for 24 h. The samples were analyzed via western blot. Representative western blots assessing uncleaved (top band) and cleaved PARP (lower band), as well as overexpressed Bcl-xL (FLAG) and β-actin (**k**). The immunoreactive bands from independent experiments, using each time independently transfected and treated cells and freshly prepared cell lysates, were quantified and PARP cleavage was calculated as in **d** (**l**). The data are plotted as mean ± SD (*N* = 5). Statistically significant differences were determined using a one-way ANOVA (*P < 0.05).

 Next, we wished to validate the importance of the IP_3_R/Bcl-xL interaction for the protective effects of Bcl-xL against Ca^2+^-dependent pro-apoptotic stimuli (Fig. 7, Fig. S7). We therefore used STS, which has been previously validated to provoke Ca^2+^-driven apoptosis [35–37]. Here, we assessed whether STS provoked apoptosis through IP_3_R-mediated Ca^2+^ elevations. First, we measured long-term Ca^2+^ dynamics in HeLa cells exposed to 0.5 µM STS for 1 hour (Fig. 7a). Using live single-cell Ca^2+^ imaging in Fura-2-AM-loaded cells, we observed that STS triggered long-lasting Ca^2+^ elevations in wild-type HeLa cells. Contrary to IP_3_R activation with physiological agonists (Fig. 1, Fig. 4), this Ca^2+^ release is rather slow on onset and prolonged over a long period of time. We then used a HeLa cell model in which all three IP_3_R isoforms have been knocked out (HeLa-3KO). In these cells, the STS-induced Ca^2+^-release events were virtually absent (Fig. 7a, b). Having validated that STS treatment in HeLa cells provoked long-lasting IP_3_R-mediated Ca^2+^ elevations, we determined whether IP_3_Rs contributed to STS-induced cell death in HeLa cells (Fig. 7c). We therefore monitored apoptotic cell death in HeLa cells exposed to 0.5 µM STS for six hours by determining the ratio of cleaved poly(ADP-ribose) polymerase (PARP) in relation to total PARP [38]. Strikingly, in wild-type HeLa, about 90% of the total PARP was converted to the cleaved form, while only 30% of the total PARP appeared in the cleaved form in HeLa-3KO cells, indicating that IP_3_Rs are crucial for STS-induced cell death in HeLa cells (Fig. 7c, d). In a more general way, this is the first time that, to our knowledge, that IP_3_Rs were directly implicated in STS-evoked pro-apoptotic Ca^2+^ flux and directly linked to cell death.

Next, using live, single-cell Ca^2+^ imaging, we studied the impact of overexpressing Bcl-xL-P2A-mCherry and Bcl-xL^K87D^-P2A-mCherry on STS-induced Ca^2+^ elevations in Fura-2-AM-loaded wild-type HeLa cells (Fig. 7e, Fig. S7a). Ca^2+^ signals were measured in mCherry-positive cells. Strikingly, Bcl-xL overexpression strongly suppressed prolonged Ca^2+^ elevations induced by 0.5 µM STS compared to empty vector-expressing cells, while Bcl-xL^K87D^ overexpression was much less effective (Fig. 7e, f, Fig. S7a, b). We then examined whether IP_3_R modulation by Bcl-xL contributed to the anti-apoptotic action of Bcl-xL (Fig. 7g). We first confirmed that the transfection of the cells with the 3xFLAG plasmids did not provoke cell death by itself (Fig. 7g, “- STS” conditions). Bcl-xL overexpression strongly suppressed PARP cleavage in wild-type HeLa cells exposed to 0.5 µM STS for 6 h compared to empty vector-expressing cells (Fig. 7g, “+ STS” conditions). In contrast, Bcl-xL^K87D^ overexpression was much less effective than wild-type Bcl-xL in suppressing PARP cleavage in wild-type HeLa cells (Fig. 7g, h). This suggests that Bcl-xL protects against STS through inhibition of IP_3_Rs, since Bcl-xL^K87D^ is much less efficient in doing so.

We then focused on IP_3_R-independent cell death mechanisms. We have shown that Bcl-xL^K87D^ binding to Bax appeared to be somewhat impaired compared to wild-type (Fig. 3c). Furthermore, the ability of Bcl-xL^K87D^ to neutralize Bax pore formation also appeared attenuated (Fig. 6). Since STS partially acts independently of IP_3_Rs and since PARP cleavage also occurs in HeLa-3KO cells, though to a lesser extent (Fig. 7d), we wanted to discriminate Bcl-xL anti-apoptotic effect between IP_3_R inhibition *versus* IP_3_R-independent processes, such as Bax inhibition. Hence, we examined the effect of Bcl-xL and Bcl-xL^K87D^ overexpression on STS-induced cell death in HeLa-3KO cells (Fig. 7i, j). Consistent with the ability of Bcl-xL to bind and neutralize Bax, we found that Bcl-xL could suppress STS-induced PARP cleavage in HeLa-3KO cells. Of particular interest and in contrast to the results obtained in wild-type HeLa cells, Bcl-xL^K87D^ was equally effective as wild-type Bcl-xL in dampening STS-induced PARP cleavage in HeLa cells lacking IP_3_Rs. This implies that although Bax binding/inhibition is somewhat affected by the K87D mutation in Bcl-xL, there is sufficient residual Bax-binding and -inhibition capacity of Bcl-xL^K87D^ to prevent cell death *in cellulo*.

 We wished to further validate that the K87D mutation does not affect the protection afforded by Bcl-xL towards IP_3_R-independent cell death triggers. Hence, we chose the BH3 mimetic venetoclax/ABT-199, a selective Bcl-2 inhibitor [16] previously established to neither interfere with the ability of Bcl-2 to inhibit IP_3_Rs nor to alter Ca^2+^ signaling [39, 40]. Venetoclax (25 µM; 24 h) triggered ~80% PARP cleavage in HeLa cells (Fig. 7k, l). The level of PARP cleavage was similar between wild-type HeLa and HeLa-3KO, thereby validating that venetoclax indeed acted in an IP_3_R-independent manner. Bcl-xL and Bcl-xL^K87D^ were equally effective in counteracting venetoclax-induced PARP cleavage by about 40–50% (Fig. 7k, l). Moreover, the anti-apoptotic effect of Bcl-xL and Bcl-xL^K87D^ was also comparable between wild-type HeLa and HeLa-3KO. These data strongly indicate that K87D mutation impairs Bcl-xL’s protective effect against IP_3_R-dependent cell death but does not significantly affects its canonical anti-apoptotic function, thereby antagonizing Bax/Bak.

### Bcl-xL protects breast cancer cells from IP_3_R-mediated cell death

**Fig. 8:**
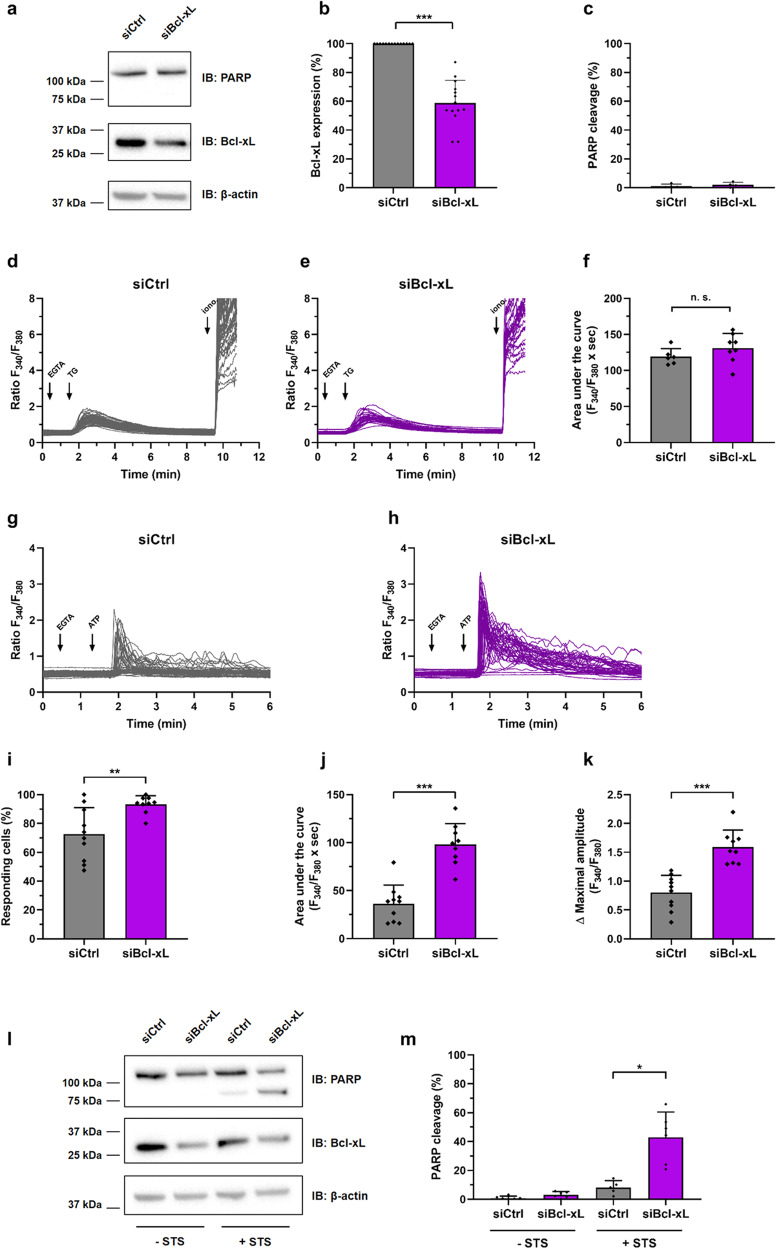
Knocking down Bcl-xL in the Bcl-xL-dependent MDA-MB-231 breast cancer cell line enhances IP_3_R-mediated Ca^2+^ release and apoptosis. MDA-MB-231 cells were transfected with either a siRNA targeting Bcl-xL (siBcl-xL, purple) or a non-target siRNA (siCtrl, black). 48 hours later, the cells were used for experiments. **a–c** Transfected cells were lysed and proteins were analyzed via western blot (IB: immunoblot). Representative western blots assessing total PARP, Bcl-xL and β-actin (**a**). Quantifications from independent experiments, using each time independently transfected cells and freshly prepared cell lysates, are shown in **b** and **c**. Data are represented as mean ± SD (*N* = 12). Statistically significant differences were determined using a *t*-test (unpaired, two-tailed, *P < 0.05). **d–k** Ca^2+^ signals obtained from Fura-2-loaded MDA-MB-231 cells. Representative traces of single cell Ca^2+^ release are shown (**d**, **e**, **g** and **h**). ER Ca^2+^ content was determined by quantifying the thapsigargin (2 µM)-releasable Ca^2+^ in the presence of EGTA (3 mM) (**d**–**f**). IP_3_R-mediated Ca^2+^ release was determined by quantifying ATP (0.5 µM)-evoked Ca^2+^ release in the presence of EGTA (3 mM) (**g**–**k**). Ionomycin (5 µM) diluted in 250 mM CaCl_2_ was added at the end of the experiment (iono.) to validate Fura-2 loading. Representative single-cell Ca^2+^ responses obtained from one well containing about 40–60 cells are shown. For each condition, 8 to 10 independent wells obtained from 3 different transfections were monitored. Area under the curves (**f**, **j**), percentages of responding cells (**i**), and maximal peak amplitudes (**k**) were calculated for each condition. Data represent mean of wells ± SD (*N* = 8 to 10), each point represents one well. Statistically significant differences were determined using a *t*-test (unpaired, two-tailed, *P < 0.05). Transfected cells were treated with 0.5 µM staurosporine (STS) for 6 h or with vehicle (DMSO). The cells were then lysed and proteins were analyzed via western blot. Representative western blots assessing uncleaved (top band) and cleaved PARP (lower band) as well as Bcl-xL and β-actin (**l**). Quantifications from independent experiments, using each time independently transfected and treated cells and freshly prepared cell lysates, are shown in **m**. Data are represented as mean ± SD (*N* = 6). Statistically significant differences were determined using a *t*-test (unpaired, two-tailed, *P < 0.05).

Finally, by knocking down Bcl-xL in a Bcl-xL-dependent cell model, we examined whether also endogenous Bcl-xL could inhibit IP_3_Rs. We used a breast cancer model, the mammary gland adenocarcinoma cell line MDA-MB-231, in which Bcl-xL is important for survival [41] and migration [42]. We transfected MDA-MB-231 cells with a siRNA targeting Bcl-xL, thereby lowering its protein levels by about 50% (Fig. 8a, b). Interestingly, Bcl-xL knock-down in MDA-MB-231 cells did not induce apoptosis by itself (Fig. 8a, c). This was important to exclude that any potential changes in Ca^2+^ signaling in cells with decreased Bcl-xL levels were a consequence of ongoing cell death rather than due to a decrease in Bcl-xL-protein levels. Using thapsigargin, we next validated in MDA-MB-231 cells that the ER Ca^2+^-store content is not altered following Bcl-xL depletion (Fig. 8d, e, f). Therefore, changes in agonist-induced Ca^2+^ signaling in Bcl-xL-depleted cells are not an indirect consequence of changes in ER Ca^2+^ loading. We then measured IP_3_R-mediated Ca^2+^ release elicited by ATP (0.5 µM) in individual MDA-MB-231 cells pre-treated with extracellular Ca^2+^ chelator EGTA, thereby ensuring that Ca^2+^ signals only arise from internal stores. Compared to the cells transfected with a non-target siRNA, the cells treated with a siRNA targeting Bcl-xL displayed a strikingly higher ATP-induced Ca^2+^ response (Fig. 8g, h). We calculated a significant increase in the number of responding cells (Fig. 8i), the area under the curve (Fig. 8j) and in the maximal peak amplitude (Fig. 8k) in MDA-MB-231 cells in which Bcl-xL-protein levels were lowered. To be certain that this effect was not due to a potential downregulation of the Bcl-xL-related Bcl-2 protein, which is a prominent inhibitor of IP_3_Rs, we analyzed the Bcl-2-protein levels via western blotting (Fig. S8). However, Bcl-2-protein levels were not decreased. Instead, Bcl-2-protein levels appeared increased, potentially as a compensatory mechanism that could help sustain the survival of the cells in which Bcl-xL was downregulated. Nevertheless, the overall Bcl-2-protein levels remained extremely low in these MDA-MB-231 cells, when benchmarked against the Bcl-2-protein levels present in OCI-LY-1 cells, a Bcl-2 dependent diffuse large B-cell lymphoma cell line. In any case, these data indicate that endogenous Bcl-xL suppresses IP_3_R activity in breast cancer cells, independently of Bcl-2 levels. To determine whether Bcl-xL could also counteract IP_3_R-mediated apoptotic Ca^2+^ release in those cells, we exposed the MDA-MB-231 cells to STS (0.5 µM) (Fig. 8l). In MDA-MB-231 cells transfected with a non-target siRNA, STS only provoked limited PARP cleavage, indicating that these cells are rather resistant to STS. However, cells treated with the siRNA against Bcl-xL displayed a prominent increase in STS-induced PARP cleavage resulting in about 50% PARP cleavage. This indicates that lowering endogenous Bcl-xL-protein levels rendered MDA-MB-231 cells very sensitive to STS-induced cell death (Fig. 8l, m). Altogether, these results reveal that endogenous Bcl-xL suppresses IP_3_R-mediated Ca^2+^ release and confers cell death protection against Ca^2+^-dependent cell death stimuli.

## Discussion

The main finding of this study is that the anti-apoptotic Bcl-xL protein functions as an inhibitor of IP_3_R channels both in intact living cells and at the single-channel level. These data challenge the presumed role of Bcl-xL as an IP_3_R-sensitizing protein [25, 26, 43]. This supposition is strongly supported by several independent lines of evidence. Molecular studies reveal that Bcl-xL targets the same regions in IP_3_Rs as Bcl-2 (e.g., LBD and Fragment 3), which are responsible for inhibition of IP_3_Rs. Further, we demonstrate that Bcl-xL, in a similar fashion to Bcl-2, possesses a lysine residue that is critical for IP_3_R binding and inhibition. The critical lysine identified in Bcl-xL (K87) spatially resembles and substitutes for the previously identified critical lysine in Bcl-2 (K17) [27]. Mutation of K87 abrogates the ability of Bcl-xL to bind and inhibit IP_3_Rs. The findings are further underpinned by single-channel recordings of IP_3_R1 channels, whose open probability is reduced upon exposure to purified Bcl-xL, but not Bcl-xL^K87D^. Moreover, by inhibiting IP_3_Rs, K87 in Bcl-xL is important for Bcl-xL’s ability to protect cells against STS, a stimulus that triggers apoptosis in an IP_3_R/Ca^2+^-dependent manner. Finally, we demonstrate that also in MDA-MB-231, a Bcl-xL-dependent breast cancer cell model, endogenous Bcl-xL inhibits IP_3_R function.

Over the past two decades, several, mainly anti-apoptotic, members of the Bcl-2-protein family, have emerged as critical modulators of Ca^2+^ homeostasis and dynamics [21]. The two most studied proteins are Bcl-2 and Bcl-xL, which are consistently reported to be localized in the ER and to control the flux through ER-resident Ca^2+^-release channels [20]. While reports suggest that Bcl-2 may also lower ER Ca^2+^-store content and thus the likelihood for pro-apoptotic Ca^2+^ transfer to mitochondria [19, 44, 45], other evidence has emerged that anti-apoptotic Bcl-2 is a direct inhibitor of Ca^2+^ flux through IP_3_Rs without markedly affecting the ER Ca^2+^-store content [46]. This results from Bcl-2 binding to IP_3_Rs [8]. Subsequent work revealed the interaction domains in both IP_3_Rs and Bcl-2 that are responsible for the complex formation. In Bcl-2, we identified the BH4 domain [23] and the C-terminal TMD [11] as critical for IP_3_R inhibition in intact cells. For IP_3_Rs, we found that the LBD [24], a stretch of 20 a.a. in the central, modulatory domain [8] and the C-terminus of IP_3_Rs [11] participate in Bcl-2 binding. Importantly, the hydrophobic cleft of Bcl-2 is not necessary for IP_3_R modulation [11, 39] and, as a result, BH3 mimetic drugs do not impact IP_3_R modulation by Bcl-2. In various cancer cell models, disrupting the complex between IP_3_Rs and Bcl-2 was even sufficient to provoke cell death through Ca^2+^ overload [47–49]. Our current model is that Bcl-2 acting via its BH4 domain inhibits IP_3_Rs by targeting the LBD and the central, modulatory domain. The occurrence of inhibition is aided by the “effective concentration” of Bcl-2 in the close proximity of IP_3_Rs as a result of the interaction between the C-terminal regions of both proteins.

Our new data demonstrate that, similarly to Bcl-2, Bcl-xL inhibits IP_3_R-mediated Ca^2+^ release by targeting precisely the same regions as the BH4 domain of Bcl-2, namely the ligand-binding domain and the central, modulatory domain. This challenges the currently accepted concept that Bcl-xL sensitizes IP_3_Rs [25, 26, 43]. We have previously proposed that the distinct modulation of IP_3_R by Bcl-2 and Bcl-xL could be due to differences in their BH4 domain [27]. Yet, *in silico* superposition of Bcl-2 and Bcl-xL indicated that the K17 residue critical for Bcl-2’s BH4 domain spatially resembled K87 of Bcl-xL [28]. Our functional results prompted us to revisit the modulation of IP_3_Rs by Bcl-xL, revealing that Bcl-xL inhibited IP_3_Rs through an interaction mediated by K87, an evolutionary conserved residue located in the BH3 domain of Bcl-xL. Moreover, similarly to Bcl-2, inhibition of IP_3_Rs by Bcl-xL was dependent on the agonist/IP_3_ concentration, whereby high agonist/IP_3_ concentrations abrogated the inhibitory effect of Bcl-xL on IP_3_Rs. This is consistent with our molecular studies showing that Bcl-xL can target the LBD, the region where IP_3_ binds. Thus, similarly to Bcl-2 [24], Bcl-xL binding to LBD might be antagonized by IP_3_.

This interaction also accounts for Bcl-xL’s protective effect against IP_3_R-mediated, Ca^2+^-driven apoptosis by using STS. Exploiting wild-type *versus* IP_3_R-knockout HeLa cells, we demonstrated that STS triggered long-lasting Ca^2+^ rises that depended on IP_3_Rs and that STS-induced cell death was for a large part driven by IP_3_Rs, though not exclusively. Bcl-xL overexpression suppressed STS-induced Ca^2+^ rises and cell death. We also observed that mutating K87 into aspartic acid in Bcl-xL also mildly impacted the ability of Bcl-xL to bind Bax and the potency of Bcl-xL to prevent Bax-pore formation. Therefore, we wanted to exclude that the impaired protection against STS-induced cell death could be due to the slightly weakened Bax-binding properties of Bcl-xL^K87D^. We therefore used the HeLa-3KO cells to exclude any IP_3_R-independent mechanism, revealing that wild-type Bcl-xL and Bcl-xL^K87D^ were equally potent in protecting HeLa cells that lacked IP_3_Rs against STS. In addition, we found that Bcl-xL and Bcl-xL^K87D^ were equally effective in protecting cells against IP_3_R-independent cell death stimuli, such as venetoclax [11, 39, 50]. Hence, this demonstrates that reduced anti-apoptotic properties of Bcl-xL^K87D^ are related to reduced inhibition of IP_3_Rs rather than non-IP_3_R-related targets such as Bax or Bak.

Our understanding of the role of Bcl-xL in Ca^2+^ signaling has been shaped by previous studies from the Foskett lab [25, 26, 43]. Therefore, the previous model is that anti-apoptotic Bcl-xL proteins enhance IP_3_R-mediated Ca^2+^ release by sensitizing the channels to IP_3_. In support of these ideas, Bcl-xL promoted IP_3_R-driven Ca^2+^ oscillations to drive mitochondrial bio-energetics and ATP production [25, 43]. In contrast to Bcl-2, Bcl-xL was proposed to bind IP_3_Rs via its hydrophobic cleft responsible for scaffolding pro-apoptotic Bcl-2-family members [26]. Thus, BH3-mimetic Bcl-xL inhibitors interfere with the ability of Bcl-xL to modulate IP_3_Rs. Moreover, the effect of Bcl-xL on IP_3_Rs was concentration-dependent with an optimal IP_3_R-sensitizing effect observed at 1 µM Bcl-xL protein. In contrast, our present data demonstrate that Bcl-xL inhibits, rather than sensitizes, IP_3_R channels. Furthermore, our live single-cell measurements show that Bcl-xL suppressed Ca^2+^ signals, even when induced by very low concentrations of agonist. By doing so, Bcl-xL seems to shift the profile of Ca^2+^ signals from long-lasting responses towards transient peaks and spontaneous oscillations (Fig. 4e). Thus, while Bcl-xL indeed increases the number of cells displaying Ca^2+^ oscillations, we argue that this is due to IP_3_R inhibition.

The reason for the discrepancy with the earlier studies is not clear, but we can exclude a number of obvious factors. First, we exclude that differences might be attributed to different cell models (here: HEK-293 and HeLa cells; [25]: DT40 cells). In the present study, we also used permeabilized DT40 cells in our electrophysiology experiments and the results we obtained were consistent with the experiments we performed in intact HEK-293 and HeLa cells. Furthermore, by sensitizing IP_3_Rs, Bcl-xL was also reported to lower ER Ca^2+^ levels in DT40 cells [51]. However, overexpressing Bcl-xL in wild-type DT40 cells did not lower the thapsigargin-induced Ca^2+^ release, indicating that in our hands Bcl-xL did not affect ER Ca^2+^-stores in these cell models (Fig. S9). We thus assume that the discrepancy with earlier studies are not related to different cell models. Second, we also controlled some other factors and validated that they can also be ruled out. i. It could be argued that in the present study, very high Bcl-xL levels were used, or that experimental conditions were not favorable to observe sensitization of Ca^2+^ release. However, in our single-channel recordings, we applied 1 µM Bcl-xL, a concentration previously reported to maximally cause IP_3_R sensitization [26]. Nevertheless, we also used even lower Bcl-xL concentrations (100–300 nM), which also inhibited IP_3_R1 single-channel openings. ii. In intact cell experiments and in IP_3_R1 single-channel recordings, we also used low concentrations of extracellular agonist and IP_3_, which should prime the system to observe IP_3_R sensitization. iii. We validated that both overexpressed Bcl-xL in living cells and purified Bcl-xL proteins used in this study are bona fide anti-apoptotic proteins and can exert anti-apoptotic functions. iv. We mainly focused on IP_3_R1 in this study as it was the IP_3_R isoform that was analyzed in depth in the original reports [25, 43]. We have not formally ruled out IP_3_R isoform-dependence in the inhibitory effect of Bcl-xL. Nevertheless, previous work indicated that all three IP_3_R isoforms could bind and were similarly sensitized by Bcl-xL in a similar fashion [25, 43]. Future work will determine whether Bcl-xL differentially impacts IP_3_R1, IP_3_R2 and IP_3_R3 channels.

At the cellular level, Bcl-xL has been shown, beyond its canonical anti-apoptotic activity, to favor cell survival by enhancing mitochondrial metabolism. For instance, Bcl-xL can interact with and promote activity of the F-type ATPase [52]. Yet, in breast cancer cells, Bcl-xL improves the metabolic capacities by more efficiently coupling the mitochondrial proton motive force with ATP production [53]. Although the involvement of Ca^2+^ signaling in those processes is still unknown, sensitization of IP_3_R by Bcl-xL has been shown to optimize mitochondrial bio-energetics, which may relate to Bcl-xL’s ability to promote Ca^2+^ flux to mitochondria [25, 54]. But if Bcl-xL does not sensitize IP_3_Rs, then how does Bcl-xL promote mitochondrial bio-energetics? In our work, we demonstrate that Bcl-xL inhibits Ca^2+^ release from the ER to the cytosol in various cell systems, including in breast cancer cells, thereby protecting the cells from IP_3_R-mediated apoptosis. Furthermore, our group has previously established that Bcl-xL inhibits the voltage-dependent anion channel 1 (VDAC1) [55]. Recently, Bcl-xL has been reported to dampen VDAC1-mediated mitochondrial Ca^2+^ uptake in breast cancer cells [56]. This mechanism has been proposed to alter mitochondrial ATP generation and increase ROS production, thereby promoting breast cancer cell migration [42]. Since Ca^2+^ transfers between the ER and the mitochondria are tightly connected, we speculate that Bcl-xL could inhibit both VDAC1 and IP_3_Rs in breast cancer cells to promote cancer malignant features.

Finally, since the interaction profile of Bcl-2 and Bcl-xL for IP_3_R binding is very similar, it is possible that peptides similar to those disrupting IP_3_R/Bcl-2 complexes, such as the Bcl-2/IP_3_R disruptor 2 (BIRD-2) [57], can affect IP_3_R/Bcl-xL complexes. Disrupting such IP_3_R/Bcl-xL complexes could therefore result in Ca^2+^-driven cell death, as observed in several Bcl-2-dependent cancer in which Bcl-2 was displaced from IP_3_Rs [22, 58] or antagonize breast cancer cell migration, a process controlled by Bcl-xL at the level of the IP_3_R [42].

Overall, this work reassesses the model and mechanism of anti-apoptotic action of Ca^2+^ signaling events modulated by Bcl-xL. In contrast to the previous model, we argue that Bcl-xL, in a similar manner to Bcl-2, inhibits IP_3_Rs and thereby can protect cells against apoptosis. Bcl-xL not only phenocopies Bcl-2 at the functional level, but also at the molecular level. This in-depth understanding of the similarities and differences in the mechanism of interaction and action of distinct anti-apoptotic Bcl-2 family members may ultimately be exploited for the design of novel therapeutics modulating apoptosis.

## Materials and methods

### Cell culture

Wild-type human cervix carcinoma cells (HeLa cells), wild-type human embryonic kidney (HEK) 293 and HEK-293 cells deficient for all three endogenous IP_3_R isoforms (3KO) stably expressing rat IP_3_R1 (HEK-rIP_3_R1) were cultured as previously described [59]. HeLa-3KO were cultured as described before [60]. Wild-type chicken lymphoblasts DT40 were cultured as previously described [61]. COS-7 and DT40 cells lacking all three IP_3_R isoforms (DT40-3KO) with ectopically expressing IP_3_R1 were cultured as described before [24]. OCI-LY-1 diffuse large B-cell lymphoma cells were cultured as previously described [39]. MDA-MB-231 breast adenocarcinoma cells were obtained from Professor P. Vangheluwe (Laboratory of Cellular Transport Systems, KU Leuven) and were bought from ATCC. MDA-MB-231 cells were cultured at 37 °C and 5% CO_2_ in Dulbecco’s modified Eagle’s medium (Sigma-Aldrich/Merck, Overijse, Belgium) supplemented with 10% fetal calf serum (Sigma-Aldrich), 1% non-essential amino acids (Gibco/Thermo Fisher Scientific, Merelbeke, Belgium), 4 mM L-glutamine, (Gibco) 100 units/ml penicillin (Gibco) and 100 µg/ml streptomycin (Gibco). All cell lines were authenticated using short tandem repeats (STR) profiling (University of Arizona Genetics Core, Tucson, Arizona). They were cultured in mycoplasma-free conditions and were monthly checked for mycoplasma infection.

### Plasmids, constructs, and protein purification

The pCMV24-3xFLAG-Bcl-xL, the pCMV24-3xFLAG-Bcl-2, the pCMV24-P2A-mCherry and the pCMV24-3xFLAG-Bcl-2-P2A-mCherry plasmids were obtained as described before [27]. The pCMV24-3xFLAG-Bcl-xL^K87D^, the pCMV24-3xFLAG-Bcl-xL-P2A-mCherry and the pCMV24-3xFLAG-Bcl-xL^K87D^-P2A-mCherry plasmids were obtained as previously described [28]. The pGEX-6p2 plasmid coding for GST-mIP_3_R1-Fragment 3 and GST-mIP_3_R1-Fragment 5b were obtained as previously described [8]. The plasmid coding for GST-mIP_3_R1-LBD construct was obtained as described before [62].

For 6xHis plasmids purification, cDNAs sequences coding for Bcl-2 and Bcl-xL were cloned in pET45b plasmids. Full-length or truncated sequences were inserted in the 6xHis-encoding reading frame. The Bcl-xL^ΔTMD^ is deleted of amino acids R209 to K233 and the Bcl-2^ΔTMD^ is deleted of amino acids L217 to K239. Following cloning of the pET45b-Bcl-xL, the K87D mutant was obtained by PCR site-directed mutagenesis as previously described [28]. All constructs were verified by sequencing (LGC Genomics, Berlin, Germany). Proteins were then purified from BL21 *Escherichia coli* as described before [24].

For GST fusion proteins purification, BL21 *Escherichia coli* were transformed and amplified as described before [62], and proteins were purified as previously described [63].

Concentration of the purified proteins was determined using BCA Protein Assay Reagent (Thermo Fisher Scientific, Merelbeke, Belgium). The purity was examined by SDS-PAGE and Coomassie blue staining of the gels with the Imperial Protein Stain reagent (ThermoFisher Scientific). Quality and integrity of the proteins were confirmed by immunoblotting with anti-GST (Cell Signaling Technology, Leiden, Netherlands; #2622) and anti Bcl-xL (Cell Signaling Technology; #2764) antibodies. Western blots were performed as previously described [59].

Bax, Bim and cBid proteins were purified from BL21 or DH5α *Escherichia coli* as extensively described before [64].

### GST-pull down assays

Two million COS-7 cells were plated in 75 cm^2^ plates. 24 h after seeding, cells were transiently transfected with 10 µg of pCMV24-3xFLAG-Bcl-xL or pCMV24-3xFLAG-Bcl-xL^K87D^ plasmids. X-tremeGene HP DNA (Roche Basel, Switzerland.) was used as a transfection reagent according to the manufacturer’s instructions. 48 h after transfection, COS cells were harvested and lysed as previously described [24], in RIPA buffer consisting of 20 mM Tris–HCl (pH 7.5), 150 mM NaCl, 1.5 mM MgCl_2_, 0.5 mM DTT, 1% Triton X-100 and protease inhibitor cocktail tablets (Roche). Cell lysates (250 µg) were pre-cleared through 1 h of incubation at 4 °C with 20 µL glutathione-Sepharose 4B beads (GE Healthcare, Diegem, Belgium). Pre-cleared lysates and GST fusion proteins were used to perform GST-pull down assays as previously described [12]. Briefly, equimolar amounts of parental GST or GST-fused fragments of IP_3_R1 (250 pmol) were incubated at 4 °C with COS lysates in 500 µL RIPA buffer. After 3 h, the GST-proteins used as bait, were immobilized on glutathione-Sepharose 4B beads (20 µL) for 2 h at 4 °C. The beads were then washed five times with RIPA buffer. The GST-complexes were eluted in 40 μl 2× LDS (Invitrogen/Thermo Fisher Scientific, Merelbeke, Belgium) supplemented with 1:200 β-mercaptoethanol by boiling for 5 min at 95 °C. Samples were analyzed via western blotting using a horseradish peroxidase-coupled anti-FLAG antibody (Sigma-Aldrich; A8592) and an anti-GST antibody (Cell Signaling Technology; #2622). Quantifications were performed using the ImageJ software (National Institutes of Health, USA).

### Co-immunoprecipitation assays

One million HeLa cells were plated in 10 cm^2^ plates. 24 h after seeding, cells were transiently transfected with 5 µg of empty pCMV24 vector or either pCMV24-3xFLAG-Bcl-xL, pCMV24-3xFLAG-Bcl-xL^K87D^ or pCMV24-3xFLAG-Bcl-2 constructs. X-tremeGene HP DNA (Roche) was used as a transfection reagent according to the manufacturer’s instructions. 48 h after transfection, HeLa cells were harvested and lysed as previously described [11]. Anti-DYKDDDDK affinity gel (BioLegend, Amsterdam, Netherlands) (30 µl) and HeLa lysates (500 µg) were used to perform co-immunoprecipitation as described before [11]. Samples were analyzed via western blotting using anti-Bax antibody (Cell Signaling Technology; #2772) or horseradish peroxidase-coupled anti-FLAG antibody (Sigma-Aldrich; A8592). We also used our homemade anti-IP_3_R1 antibody Rbt03 [65].

### Gene knockdown

300,000 MDA-MB-231 cells were plated in six-well plates. 24 h after seeding, cells were transfected with 500 nM siRNA targeting human Bcl-xL’s mRNA (hs.Ri.BCL2L1.13.1, Integrated DNA Technologies, Leuven, Belgium) or 500 nM non-targeting control pool siRNA (Dharmacon/Horizon Discovery, Cambridge, United Kingdom). Lipofectamine RNAiMAX (Invitrogen) was used as a transfection reagent according to the manufacturer’s instructions. At 48 h post-transfection, the cells were used for experiments. Effective gene knockdown was confirmed via western blotting using anti-Bcl-xL (Cell Signaling Technology; #2764) and anti-β-actin (Sigma-Aldrich; A5441) antibodies. An HRP-coupled anti-Bcl-2 antibody (Santa Cruz Biotechnology, Heidelberg, Germany; sc-7382 HRP) was used to assess for changes in Bcl-2-protein levels after knockdown of Bcl-xL.

### Apoptosis induction

300,000 HeLa cells were plated in six-well plates. 24 h after seeding, cells were transiently transfected with 1 µg of empty pCMV24 vector, pCMV24-3xFLAG-Bcl-xL or pCMV24-3xFLAG-Bcl-xL^K87D^ constructs. X-tremeGene HP DNA (Roche) was used as a transfection reagent according to the manufacturer’s instructions. 48 h after transfection, HeLa cells were treated with 0.5 µM staurosporine (Sigma-Aldrich) for 6 h or with 25 µM venetoclax (Cayman Chemical/Sanbio, Uden, Netherlands). DMSO (Invitrogen) was used as vehicle. Cells were then harvested and lysed as previously described [11]. Apoptosis was monitored via western-blotting with an anti-PARP1 antibody (Cell Signaling Technology, #9532) that detected both cleaved and uncleaved PARP. PARP cleavage was calculated as the ratio of cleaved PARP over total PARP. Quantifications were performed using the ImageJ software (National Institutes of Health, USA). We also used anti-Bcl-xL (Cell Signaling Technology; #2764), anti-β-actin (Sigma-Aldrich; A5441) and anti-vinculin (Sigma-Aldrich; V9131) antibodies.

### Cell populations Ca^2+^ measurements

60,000 HEK-rIP_3_R1 cells were plated in 96-well plates. 24 h after seeding, cells were transiently transfected with 0.05 µg of pCMV24-P2A-mCherry vector or pCMV24-3xFLAG-Bcl-xL-P2A-mCherry construct. X-tremeGene HP DNA (Roche) was used as a transfection reagent according to the manufacturer’s instructions. 48 h after transfection, HEK-rIP3R1 cells were loaded with Fura-2-AM (AnaSpec/Kaneka Eurogentec, Seraing, Belgium) and Ca^2+^ imaging was monitored using a FlexStation 3 microplate reader (Molecular Devices, Sunnyvale, CA, USA) as previously described [66]. Trypsin from porcine pancreas (Sigma-Aldrich) was used to elicit IP_3_R-mediated Ca^2+^ release. 

### Single-cell Ca^2+^ imaging

20,000 HeLa, 50,000 HEK-293 or 50 000 MDA-MB-231 cells were plated in four-chamber 35-mm dishes. 24 h after seeding, cells were transiently transfected with 0.25 µg of pCMV24-P2A-mCherry, pCMV24-3xFLAG-Bcl-2-P2A-mCherry, pCMV24-3xFLAG-Bcl-xL-P2A-mCherry or pCMV24-3xFLAG-Bcl-xL^K87D^-P2A-mCherry constructs. X-tremeGene HP DNA (Roche) was used as a transfection reagent according to the manufacturer’s instructions. 48 h after transfection, the cells were loaded with Fura-2-AM (AnaSpec) or Fluo-4-AM (Invitrogen) and Ca^2+^ imaging was performed using an Axio Observer Z1 fluorescent microscope (Zeiss, Jena, Germany) as previously described [27]. Data were plotted either as F_340_/F_380_ ratio, [Ca^2+^], or as normalized (F − F_0_)/F_0_ or F/F_0_ whereby F = F_340_/F_380_ at different time points and F_0_ = F_340_/F_380_ at the start of the experiment. EGTA (Sigma-Aldrich) was used to chelate extracellular Ca^2+^. ATP (Sigma-Aldrich) and carbachol (Sigma-Aldrich) were used to elicit IP_3_R-mediated Ca^2+^ release. Thapsigargin (Alomone Labs, Jerusalem, Israel), an irreversible inhibitor of sarco/endoplasmic reticulum Ca^2+^ ATPases, was used to assess ER Ca^2+^-store content. Ionomycin (Alomone Labs), a Ca^2+^ ionophore, was used to assess the total intracellular Ca^2+^ and validate adequate loading of fluorescent Ca^2+^ indicators. Ca^2+^ traces were analyzed with the Excel (Microsoft, Redmond, WA, USA) and Prism (GraphPad, San Diego, CA, USA) softwares. The number of non-responding cells was determined, whereby non-responding cells were defined as cells in which the maximal fluorescence signal measured after agonist stimulation do not exceed the baseline value + SEM. The Δ maximal amplitude and area under the curve were analyzed in the responding cells. The baseline was calculated as the average fluorescence between EGTA and agonist addition. The Δ maximal amplitude was calculated by subtracting the baseline from the maximal response value to the agonist. The area under the curve was calculated by integrating the responses to agonist after subtracting the baseline.

### Electrophysiology

Isolated nuclei from DT40-3KO cells stably transfected with rat IP_3_R1 were prepared by homogenization as previously described [67]. Patch-clamp experiments were performed as described before [24]. Purified recombinant proteins 6xHis-Bcl-xL, 6xHis-Bcl-xL^ΔTMD^, 6xHis-Bcl-xL^K87D^ and 6xHis-Bcl-2^ΔTMD^ were used during electrophysiology experiments.

### Surface plasmon resonance

The following peptides (purity >80%) were obtained from LifeTein (South Plainfield, NJ, USA) and dissolved in dimethyl sulfoxide to prepare 10 mM stock solutions.

Biotin-BH4-Bcl-2: biotin-RTGYDNREIVMKYIHYKLSQRGYEW;

Biotin-BH4-Bcl-2-scramble: biotin-WYEKQRSLHGIMYYVIEDRNTKGYR;

Biotin-BH4-Bcl-xL: biotin-MSQSNRELVVDFLSYKLSQKGYSW;

Biotin-BH4-Bcl-xL-scramble: biotin-WYSKQRSLSGLVMYVLEDKNSQFS;

Biotinylated peptides (200 ng), immobilized on streptavidin-sensor chips, and purified GST-LBD proteins, applied as analyte, were used to perform SPR assays as described before [27].

### Mammalian protein–protein interaction trap

MAPPIT experiments were performed as previously described [28]. Briefly, the Fragment 3 of mIP_3_R1 was cloned in a pSEL+2L bait vector, downstream of a chimeric cytokine receptor (Fragment 3 bait), consisting of the extracellular domain of the erythropoietin receptor fused to the transmembrane and cytosolic part of the leptin receptor. Bcl-xL or Bcl-xL^K87D^ was cloned in a in the pMG1-GW plasmid, downstream of a part of the glycoprotein 130 receptor (Bcl-xL or Bcl-xL^K87D^ prey). The interaction between Fragment 3 and Bcl-xL or Bcl-xL^K87D^ is detected by a luciferase reporter assay driven by a STAT-responsive promoter, since the functional complementation of the chimeric cytokine receptor results in ligand-dependent downstream STAT signaling. We also used the SV40 large antigen T as an irrelevant prey to monitor the signal representing the non-specific binding to Fragment 3. As an extra negative control, binding of the chimeric cytokine receptor without the Fragment 3 fragment (no bait) to the two Bcl-xL preys was also assessed.

### Liposome permeabilization assay

Liposomes encapsulating the fluorophore 8-aminonaphthalene-1,3,6-trisulfonic acid (ANTS) and the collisional quencher *p*-xylene-bis-pyridinium bromide (DPX) were prepared as previously described [64]. Purified Bcl-xL, cBid (20 nM), Bim (20 nM) or Bax (100 nM) proteins were then incubated with the liposomes (0.04 mg/ml) and ANTS/DPX release was measured as described before [34].

### CD spectrum and thermal ramping

The experiments were performed as described in [68]. CD spectra were recorded using a J‐1500 spectropolarimeter (Jasco, Easton, MD, USA) equipped with a Peltier element for temperature control and a six‐position cuvette holder. Proteins were dialyzed in 5 mM MOPS pH:7.5; 5 mM NaCl, for 15 h, at 4 °C; 3× changes; constant stirring. Aggregated material was removed by centrifugation (20,000 *g*; 15 min; 4 °C) before protein concentration was determined on a Nanodrop instrument (280 nm; 2000 series; Thermo). The molecular extinction coefficient and molecular weight for A280 analysis was determined using the Expasy server (http://web.expasy.org/protparam/). Wavelength scan measurements (190–260 nm) were performed with 15 μM protein, at 20 °C; in 5 mM MOPS pH 7.5; 5 mM NaCl, using 1 mm quartz cuvettes (Hellma, Müllheim, Germany); data pitch: 0.5 nm; bandwidth: 1 nm; scanning speed: 50 nm·min^−1^; DIT: 0.5 s; accumulation: 3. Variable temperature measurements (15–90 °C) were performed with 15 μM protein; in 5 mM MOPS pH 7.5; 5 mM NaCl, using 1 mm quartz cuvettes (Hellma); interval 0.5 °C; gradient 1 °C· min^−1^; DIT: 0.5 s; bandwidth: 1 nm. Data were analyzed using the spectra analysis v.2 software (Jasco); Tm_app_ were derived by acquiring the first derivatives of the melting curves, using the calculus function of the Origin 7 software (GE Healthcare).

### Microscale thermophoresis

Both purified 6xHis-Bcl-xL protein and 6xHis-Bcl-xL^K87D^ were fluorescently labeled using the Monolith His-Tag Labeling Kit RED-tris-NTA 2nd Generation (Nano Temper Technologies, Munich, Germany) and binding affinities were evaluated using microscale thermophoresis. Concentration of 6xHis-Bcl-xL and 6xHis-Bcl-xL^K87D^ was kept constant at 50 nM, whereas the GST-LBD, GST-Fragment 3, GST-Fragment 5b and GST-control proteins were titrated down from 15 µM to 5 nM. Measurements were performed in steady-state conditions using premium capillaries and subsequently recorded on a Monolith NT automated instrument (Nano Temper Technologies) with a pico-red laser channel at 5% excitation power and “medium” MST power. All experiments were repeated three times for each measurement. The Prism software (GraphPad) was used to plot the data points, fit a nonlinear regression curve and calculate the dissociation constant (*K*_d_) for each condition.

### Confocal microscopy

100,000 HeLa cells were plated on 18 mm diameter coverslips coated with 0.1 mg/ml poly-L-lysin (Sigma-Aldrich) in 12-well plates and cultured as described above. Twenty-four hours later, the cells were co-transfected with 1 µg pCMV24-3xFLAG-Bcl-xL or pCMV24-3xFLAG-Bcl-xL^K87D^ along with 1 µg of plasmid coding for an ER-targeted RFP or a mitochondria-targeted RFP (mito-RFP). The RR-RFP and mito-RFP were a gift from Professor P. Agostinis (Laboratory of Cell Death Research & Therapy, KU Leuven). X-tremeGene HP DNA (Roche) was used as a transfection reagent according to the manufacturer’s instructions. Twenty-four hours after transfection, the cells were fixated in 4% paraformaldehyde for 10 min and permeabilized in 0.3% Triton X-100 for 15 min at room temperature. The blocking was performed with 5% bovine serum albumin (Sigma-Aldrich) in PBS for 30 min at room temperature. Primary antibodies diluted in blocking buffer were applied overnight at 4 °C and secondary antibodies diluted in blocking buffer were applied for 1 h at room temperature. Mouse anti-FLAG antibodies (Sigma-Aldrich; F3165) were used as primary antibodies while mouse IgG1s (Dako/Agilent Technologies, Heverlee, Belgium) were used for negative controls, at the same concentration (5 µg/ml). Alexa Fluor 488-coupled goat anti-mouse (Molecular Probes/Thermo Fisher Scientific, Merelbeke, Belgium; A-11017) were used as secondary antibodies (4 µg/ml). The coverslips were eventually mounted on glass slides with the Faramount Mounting Medium Ready (Dako/Agilent Technologies). The cells were imaged using an Axiovert 100 M LSM 510 confocal microscope (Zeiss). Image processing was performed with the ImageJ software (National Institutes of Health, USA) and colocalization was measured with the JACoP plugin [69].

### Phylogenetic analysis

Conserved amino acid motifs were calculated using the MEME suite tool. The multiple sequence alignment of the motifs was done in MEGA7. Orthologous sequences for the alignment were selected based on the phylogenetic analysis, performed in RAxML tool with the PROTGAMMALG matrix (Supp. file 1).

### Statistical analysis

The Prism software (GraphPad) was used for statistical analysis. Data are expressed as mean ± SEM or SD. Two-tailed Student’s *t* tests were used to compare two conditions and repeated-measure ANOVA with Bonferroni post-tests were performed when comparing three or more conditions. For small sample sizes, non-parametric tests were performed. Statistically significant differences were considered at *P* < 0.05 (*), *P* < 0.01 (**) and *P* < 0.005 (***).

## Supplementary information

Supplemental Figure Legends

Supplemental Figure 1

Supplemental Figure 2

Supplemental Figure 3

Supplemental Figure 4

Supplemental Figure 5

Supplemental Figure 6

Supplemental Figure 7

Supplemental Figure 8

Supplemental Figure 9

Supplementary file 1: orthologous sequence selection

## References

[1] Foskett JK, White C, Cheung KH, Mak DO (2007). Inositol trisphosphate receptor Ca^2+^ release channels. Physiol Rev.

[2] Hamada K, Mikoshiba K (2020). IP_3_ receptor plasticity underlying diverse functions. Annu Rev Physiol.

[3] Berridge MJ (2016). The inositol trisphosphate/calcium signaling pathway in health and disease. Physiol Rev.

[4] Bootman MD, Bultynck G. Fundamentals of cellular calcium signaling: a primer. In: Bultynck G, Bootman MD, Berridge MJ, Stutzmann GE, editors. Calcium signaling, Second Edition. New York: Cold Spring Harbor Laboratory Press; 2019. 1–16.

[5] Prole DL, Taylor CW (2016). Inositol 1,4,5-trisphosphate receptors and their protein partners as signalling hubs. J Physiol.

[6] Parys JB, Vervliet T (2020). New Insights in the IP_3_ receptor and its regulation. Adv Exp Med Biol.

[7] Yoshikawa F, Iwasaki H, Michikawa T, Furuichi T, Mikoshiba K (1999). Trypsinized cerebellar inositol 1,4,5-trisphosphate receptor. Structural and functional coupling of cleaved ligand binding and channel domains. J Biol Chem.

[8] Rong YP, Aromolaran AS, Bultynck G, Zhong F, Li X, McColl K (2008). Targeting Bcl-2-IP_3_ receptor interaction to reverse Bcl-2’s inhibition of apoptotic calcium signals. Mol Cell.

[9] Lee B, Vermassen E, Yoon SY, Vanderheyden V, Ito J, Alfandari D (2006). Phosphorylation of IP_3_R1 and the regulation of [Ca^2+^]i responses at fertilization: a role for the MAP kinase pathway. Development..

[10] Harr MW, Rong Y, Bootman MD, Roderick HL, Distelhorst CW (2009). Glucocorticoid-mediated inhibition of Lck modulates the pattern of T cell receptor-induced calcium signals by down-regulating inositol 1,4,5-trisphosphate receptors. J Biol Chem.

[11] Ivanova H, Ritaine A, Wagner L, Luyten T, Shapovalov G, Welkenhuyzen K (2016). The trans-membrane domain of Bcl-2alpha, but not its hydrophobic cleft, is a critical determinant for efficient IP_3_ receptor inhibition. Oncotarget..

[12] Monaco G, Beckers M, Ivanova H, Missiaen L, Parys JB, De Smedt H (2012). Profiling of the Bcl-2/Bcl-X_L_-binding sites on type 1 IP_3_ receptor. Biochem Biophys Res Commun.

[13] Aouacheria A, Baghdiguian S, Lamb HM, Huska JD, Pineda FJ, Hardwick JM (2017). Connecting mitochondrial dynamics and life-or-death events via Bcl-2 family proteins. Neurochem Int.

[14] Singh R, Letai A, Sarosiek K (2019). Regulation of apoptosis in health and disease: the balancing act of BCL-2 family proteins. Nat Rev Mol Cell Biol.

[15] Adams JM, Cory S (2018). The BCL-2 arbiters of apoptosis and their growing role as cancer targets. Cell Death Differ.

[16] Montero J, Letai A (2018). Why do BCL-2 inhibitors work and where should we use them in the clinic?. Cell Death Differ.

[17] Kale J, Osterlund EJ, Andrews DW (2018). BCL-2 family proteins: changing partners in the dance towards death. Cell Death Differ.

[18] Kalkavan H, Green DR (2018). MOMP, cell suicide as a BCL-2 family business. Cell Death Differ.

[19] Scorrano L, Oakes SA, Opferman JT, Cheng EH, Sorcinelli MD, Pozzan T (2003). BAX and BAK regulation of endoplasmic reticulum Ca^2+^: a control point for apoptosis. Science..

[20] Ivanova H, Vervliet T, Monaco G, Terry LE, Rosa N, Baker MR (2020). Bcl-2-protein family as modulators of IP_3_ receptors and other organellar Ca^2+^ channels. Cold Spring Harb Perspect Biol.

[21] Vervliet T, Parys JB, Bultynck G (2016). Bcl-2 proteins and calcium signaling: complexity beneath the surface. Oncogene..

[22] Distelhorst CW, Bootman MD. Creating a new cancer therapeutic agent by targeting the interaction between Bcl-2 and IP_3_ receptors. In: Bultynck G, Bootman MD, Berridge MJ, Stutzmann GE, editors. Calcium signaling, Second Edition. New York: Cold Spring Harbor Laboratory Press; 2019. 463–78. 10.1101/cshperspect.a035196 PMC6719601 31110129

[23] Rong YP, Bultynck G, Aromolaran AS, Zhong F, Parys JB, De Smedt H (2009). The BH4 domain of Bcl-2 inhibits ER calcium release and apoptosis by binding the regulatory and coupling domain of the IP_3_ receptor. Proc Natl Acad Sci USA.

[24] Ivanova H, Wagner LE, Tanimura A, Vandermarliere E, Luyten T, Welkenhuyzen K (2019). Bcl-2 and IP_3_ compete for the ligand-binding domain of IP_3_Rs modulating Ca^2+^ signaling output. Cell Mol Life Sci.

[25] White C, Li C, Yang J, Petrenko NB, Madesh M, Thompson CB (2005). The endoplasmic reticulum gateway to apoptosis by Bcl-X_L_ modulation of the InsP_3_R. Nat Cell Biol.

[26] Yang J, Vais H, Gu W, Foskett JK (2016). Biphasic regulation of InsP3 receptor gating by dual Ca^2+^ release channel BH3-like domains mediates Bcl-xL control of cell viability. Proc Natl Acad Sci USA.

[27] Monaco G, Decrock E, Akl H, Ponsaerts R, Vervliet T, Luyten T (2012). Selective regulation of IP_3_-receptor-mediated Ca^2+^ signaling and apoptosis by the BH4 domain of Bcl-2 versus Bcl-Xl. Cell Death Differ.

[28] Vervliet T, Lemmens I, Vandermarliere E, Decrock E, Ivanova H, Monaco G (2015). Ryanodine receptors are targeted by anti-apoptotic Bcl-XL involving its BH4 domain and Lys87 from its BH3 domain. Sci Rep.

[29] Alzayady KJ, Wang L, Chandrasekhar R, Wagner LE, Van Petegem F, Yule DI (2016). Defining the stoichiometry of inositol 1,4,5-trisphosphate binding required to initiate Ca^2+^ release. Sci Signal.

[30] Eyckerman S, Verhee A, der Heyden JV, Lemmens I, Ostade XV, Vandekerckhove J (2001). Design and application of a cytokine-receptor-based interaction trap. Nat Cell Biol.

[31] Sauve R, Diarra A, Chahine M, Simoneau C, Morier N, Roy G (1991). Ca^2+^ oscillations induced by histamine H1 receptor stimulation in HeLa cells: Fura-2 and patch clamp analysis. Cell Calcium.

[32] Okuda A, Furuya K, Kiyohara T (2003). ATP-induced calcium oscillations and change of P2Y subtypes with culture conditions in HeLa cells. Cell Biochem Funct.

[33] Jeong SY, Gaume B, Lee YJ, Hsu YT, Ryu SW, Yoon SH (2004). Bcl-x_L_ sequesters its C-terminal membrane anchor in soluble, cytosolic homodimers. EMBO J.

[34] Bogner C, Kale J, Pogmore J, Chi X, Shamas-Din A, Fradin C (2020). Allosteric regulation of BH3 proteins in Bcl-xL complexes enables switch-like activation of Bax. Mol Cell.

[35] Assefa Z, Bultynck G, Szlufcik K, Nadif Kasri N, Vermassen E, Goris J (2004). Caspase-3-induced truncation of type 1 inositol trisphosphate receptor accelerates apoptotic cell death and induces inositol trisphosphate-independent calcium release during apoptosis. J Biol Chem.

[36] Szalai G, Krishnamurthy R, Hajnoczky G (1999). Apoptosis driven by IP_3_-linked mitochondrial calcium signals. EMBO J.

[37] De Stefani D, Bononi A, Romagnoli A, Messina A, De Pinto V, Pinton P (2012). VDAC1 selectively transfers apoptotic Ca^2+^ signals to mitochondria. Cell Death Differ.

[38] Virag L, Robaszkiewicz A, Rodriguez-Vargas JM, Oliver FJ (2013). Poly(ADP-ribose) signaling in cell death. Mol Asp Med.

[39] Vervloessem T, Ivanova H, Luyten T, Parys JB, Bultynck G (2017). The selective Bcl-2 inhibitor venetoclax, a BH3 mimetic, does not dysregulate intracellular Ca^2+^ signaling. Biochim Biophys Acta Mol Cell Res.

[40] Vervloessem T, Kerkhofs M, La Rovere RM, Sneyers F, Parys JB, Bultynck G (2018). Bcl-2 inhibitors as anti-cancer therapeutics: the impact of and on calcium signaling. Cell Calcium.

[41] Soderquist RS, Crawford L, Liu E, Lu M, Agarwal A, Anderson GR (2018). Systematic mapping of BCL-2 gene dependencies in cancer reveals molecular determinants of BH3 mimetic sensitivity. Nat Commun.

[42] Bessou M, Lopez J, Gadet R, Deygas M, Popgeorgiev N, Poncet D (2020). The apoptosis inhibitor Bcl-xL controls breast cancer cell migration through mitochondria-dependent reactive oxygen species production. Oncogene..

[43] Li C, Wang X, Vais H, Thompson CB, Foskett JK, White C (2007). Apoptosis regulation by Bcl-x_L_ modulation of mammalian inositol 1,4,5-trisphosphate receptor channel isoform gating. Proc Natl Acad Sci USA.

[44] Pinton P, Ferrari D, Magalhaes P, Schulze-Osthoff K, Di Virgilio F, Pozzan T (2000). Reduced loading of intracellular Ca^2+^ stores and downregulation of capacitative Ca^2+^ influx in Bcl-2-overexpressing cells. J Cell Biol.

[45] Pinton P, Ferrari D, Rapizzi E, Di Virgilio F, Pozzan T, Rizzuto R (2001). The Ca^2+^ concentration of the endoplasmic reticulum is a key determinant of ceramide-induced apoptosis: significance for the molecular mechanism of Bcl-2 action. EMBO J.

[46] Chen R, Valencia I, Zhong F, McColl KS, Roderick HL, Bootman MD (2004). Bcl-2 functionally interacts with inositol 1,4,5-trisphosphate receptors to regulate calcium release from the ER in response to inositol 1,4,5-trisphosphate. J Cell Biol.

[47] Zhong F, Harr MW, Bultynck G, Monaco G, Parys JB, De Smedt H (2011). Induction of Ca^2+^-driven apoptosis in chronic lymphocytic leukemia cells by peptide-mediated disruption of Bcl-2-IP_3_ receptor interaction. Blood..

[48] Akl H, Monaco G, La Rovere R, Welkenhuyzen K, Kiviluoto S, Vervliet T (2013). IP_3_R2 levels dictate the apoptotic sensitivity of diffuse large B-cell lymphoma cells to an IP_3_R-derived peptide targeting the BH4 domain of Bcl-2. Cell Death Dis.

[49] Bittremieux M, La Rovere RM, Akl H, Martines C, Welkenhuyzen K, Dubron K (2019). Constitutive IP_3_ signaling underlies the sensitivity of B-cell cancers to the Bcl-2/IP_3_ receptor disruptor BIRD-2. Cell Death Differ.

[50] Jakubowska MA, Kerkhofs M, Martines C, Efremov DF, White C, Gerasimenko JV, Gerasimenko OV (2019). ABT-199 (Venetoclax), a BH3-mimetic Bcl-2 inhibitor, does not cause Ca^2+^-signalling dysregulation or toxicity in pancreatic acinar cells. Br J Pharmacol.

[51] Eckenrode EF, Yang J, Velmurugan GV, Foskett JK, White C (2010). Apoptosis protection by Mcl-1 and Bcl-2 modulation of inositol 1,4,5-trisphosphate receptor-dependent Ca^2+^ signaling. J Biol Chem.

[52] Alavian KN, Li H, Collis L, Bonanni L, Zeng L, Sacchetti S (2011). Bcl-xL regulates metabolic efficiency of neurons through interaction with the mitochondrial F1FO ATP synthase. Nat Cell Biol.

[53] Lucantoni F, Salvucci M, Dussmann H, Lindner AU, Lambrechts D, Prehn JHM (2020). BCL(X)L and BCL2 increase the metabolic fitness of breast cancer cells: a single-cell imaging study. Cell Death Differ.

[54] Williams A, Hayashi T, Wolozny D, Yin B, Su TC, Betenbaugh MJ (2016). The non-apoptotic action of Bcl-xL: regulating Ca^2+^ signaling and bioenergetics at the ER-mitochondrion interface. J Bioenerg Biomembr.

[55] Monaco G, Decrock E, Arbel N, van Vliet AR, La Rovere RM, De, Smedt H (2015). The BH4 domain of anti-apoptotic Bcl-XL, but not that of the related Bcl-2, limits the voltage-dependent anion channel 1 (VDAC1)-mediated transfer of pro-apoptotic Ca^2+^ signals to mitochondria. J Biol Chem.

[56] Huang H, Hu X, Eno CO, Zhao G, Li C, White C (2013). An interaction between Bcl-xL and the voltage-dependent anion channel (VDAC) promotes mitochondrial Ca^2+^ uptake. J Biol Chem.

[57] Kerkhofs M, Bultynck G, Vervliet T, Monaco G (2019). Therapeutic implications of novel peptides targeting ER-mitochondria Ca^2+^-flux systems. Drug Discov Today.

[58] Kerkhofs M, Vervloessem T, Bittremieux M, Bultynck G (2019). Recent advances in uncovering the mechanisms contributing to BIRD-2-induced cell death in B-cell cancer cells. Cell Death Dis.

[59] Luyten T, Welkenhuyzen K, Roest G, Kania E, Wang L, Bittremieux M (2017). Resveratrol-induced autophagy is dependent on IP_3_Rs and on cytosolic Ca^2+^. Biochim Biophys Acta Mol Cell Res.

[60] Ando H, Hirose M, Mikoshiba K (2018). Aberrant IP_3_ receptor activities revealed by comprehensive analysis of pathological mutations causing spinocerebellar ataxia 29. Proc Natl Acad Sci USA.

[61] Bultynck G, Kiviluoto S, Henke N, Ivanova H, Schneider L, Rybalchenko V (2012). The C terminus of Bax inhibitor-1 forms a Ca^2+^-permeable channel pore. J Biol Chem.

[62] Bultynck G, Szlufcik K, Kasri NN, Assefa Z, Callewaert G, Missiaen L (2004). Thimerosal stimulates Ca^2+^ flux through inositol 1,4,5-trisphosphate receptor type 1, but not type 3, via modulation of an isoform-specific Ca^2+^-dependent intramolecular interaction. Biochem J.

[63] Sienaert I, Missiaen L, De Smedt H, Parys JB, Sipma H, Casteels R (1997). Molecular and functional evidence for multiple Ca^2+^-binding domains in the type 1 inositol 1,4,5-trisphosphate receptor. J Biol Chem.

[64] Kale J, Chi X, Leber B, Andrews D (2014). Examining the molecular mechanism of bcl-2 family proteins at membranes by fluorescence spectroscopy. Methods Enzymol.

[65] Parys JB, de Smedt H, Missiaen L, Bootman MD, Sienaert I, Casteels R (1995). Rat basophilic leukemia cells as model system for inositol 1,4,5-trisphosphate receptor IV, a receptor of the type II family: functional comparison and immunological detection. Cell Calcium.

[66] Decuypere JP, Welkenhuyzen K, Luyten T, Ponsaerts R, Dewaele M, Molgo J (2011). Ins(1,4,5)P3 receptor-mediated Ca^2+^ signaling and autophagy induction are interrelated. Autophagy.

[67] Wagner LE, Yule DI (2012). Wagner LE 2nd, Yule DI. Differential regulation of the InsP_3_ receptor type-1 and -2 single channel properties by InsP_3_, Ca^2+^ and ATP. J Physiol.

[68] Monaco G, La Rovere R, Karamanou S, Welkenhuyzen K, Ivanova H, Vandermarliere E (2018). A double point mutation at residues Ile14 and Val15 of Bcl-2 uncovers a role for the BH4 domain in both protein stability and function. FEBS J.

[69] Bolte S, Cordelieres FP (2006). A guided tour into subcellular colocalization analysis in light microscopy. J Microsc.

