# Supplementary material for: Bcl-xL acts as an inhibitor of IP3R channels, thereby antagonizing Ca2+-driven apoptosis
Source: Cell Death Differ. 2021 Nov 8;29(4):788–805. doi: 10.1038/s41418-021-00894-w (PMC8990011; doi:10.1038/s41418-021-00894-w)
Supplement: Supplementary file 1 — Supplemental Figure Legends [file 41418_2021_894_MOESM1_ESM.docx]

***Fig. S1: Bcl-xL overexpression suppresses trypsin-evoked IP_3_R-mediated Ca^2+^ release in living cells.***

Ca^2+^ measurements obtained from Fura-2-loaded HEK-3KO-rIP_3_R1 cells transfected with a Bcl-xL-encoding vector (pCMV24-3xFLAG-Bcl-xL-P2A-mCherry; green) or an empty vector (pCMV24-mCherry; black). Representative traces of Ca^2+^ release are shown in **a** and **c**. ER Ca^2+^ response is elicited by addition of 0.1 µM (**a, b**) or 1 µM trypsin (**c, d**). 3 mM EGTA was added 60 seconds after the beginning of the measurement to chelate extracellular Ca^2+^. 5 µM ionomycin (not shown) diluted in 250 mM CaCl_2_ was added at the end of the experiment to trigger a high Ca^2+^ release and confirm the cells are equally loaded with Fura-2. Calcium responses were measured in a cell population. Traces represent mean ± SEM of triplicate samples of one representative experiment (**a**, **c**). Areas under the curve were calculated from the Ca^2+^ traces obtained upon 0.1 µM (**b**) or 1 µM trypsin (**d**) stimulation. Data are represented as mean ± SEM (*N* = 3). Statistically significant differences were determined using a *t*-test (unpaired, two-tailed, **P* < 0.05).

***Fig. S2: Control immunoblots for GST pull-downs experiments.***

GST-pull down experiment for assessing the binding of 3xFLAG-Bcl-xL or 3xFLAG-Bcl-xL^K87D^ from COS-7 cell lysates to GST-fused IP_3_R1 fragments. The pull-down eluates were subjected to western blot. Anti-FLAG stainings were performed to detect binding of 3xFLAG-Bcl-xL proteins (Fig. 2c, Fig. 3f) and anti-GST stainings were performed to validate the GST-IP3R1 fragments used in the experiment (panel **a** is control for Fig. 2c and panel **b** is control for Fig. 3f). The GST-IP3R1-LBD and the GST-IP3R1-Fragment 3 have a predicted molecular weight of 95 kDa and 104 kDa, respectively. * indicates the full-length proteins, whereby the lower bands in the GST-LBD lane represent degradation products. PD: pull down; IB: immunoblot.

***Fig. S3: The K87D mutation in Bcl-xL does not affect the subcellular localization of Bcl-xL***

Immunofluorescence of HeLa cells transfected to overexpress a mitochondria-targeted RFP (mito-RFP) (**a**, **b**, **i**, **j**) or an ER-targeted RFP (ER-RFP) (**c**, **d**, **k**, **l**) as well as 3xFLAG-Bcl-xL (**a**, **c**, **i**, **k**) or 3xFLAG-Bcl-xL^K87D^ (**b**, **d**, **j**, **l**). The cells were labeled with anti-FLAG antibodies (**a**, **b**, **c**, **d**). The stainings were imaged with a confocal microscope using 63x magnification with a 2x zoom. About 20 cells per condition were imaged from two independent experiments to quantify colocalization. Pearson’s coefficient (**e**, **f**) and Manders’ M1 coefficient (**g**, **h**) were calculated for each individual cell and for each condition. Each data point represents one single cell and the bars represent the mean (*N* = 18 to 22). Statistically significant differences were determined using a *t*-test (unpaired, two-tailed, **P* < 0.05). For negative controls, non-specific mouse IgG1 were used instead of anti-FLAG antibodies at the same concentration (**i**, **j**, **k**, **l**).

***Fig. S4: Purified 6xHis-Bcl-xL******^K87D^ does not bind to GST***

Binding curves showing the interaction of purified 6xHis-Bcl-xL^K87D^ with titrated GST. Concentration of the 6xHis-Bcl-xL was kept constant at 25 nM, whereas the GST proteins were titrated down from 15 µM to 5 nM. The unit of the left axis (ΔF_norm_) is a ratio of normalized fluorescence. Data points represent mean ± SD from triplicate measurements.

***Fig. S5: Neither Bcl-xL nor Bcl-xL^K87D^ overexpression alters*** ***ER Ca^2+^-store content in single HeLa cells.***

Calcium measurements obtained from Fura-2-loaded Hela cells transfected with an empty vector (black), 3xFLAG-Bcl-xL-P2A-mCherry (green) or 3xFLAG-Bcl-xL^K87D^-P2A-mCherry (red). ER Ca^2+^ loading was determined by measuring the response to thapsigargin (1 µM) after addition of the extracellular Ca^2+^ buffer EGTA (3 mM). Ionomycin (5 µM) diluted in 250 mM CaCl_2_ was added at the end of the experiment (not shown) to trigger a high Ca^2+^ release and confirm all the cells are equally loaded with Fura-2. **a** Ca^2+^ Traces represent mean ± SEM of one representative experiment. Two wells and about 20-30 cells/well were monitored for each condition. **b** Area under the curve were calculated from the Ca^2+^ traces. Data represent mean ± SEM of three experiments (*N* = 3). Statistically significant differences were determined using a *t*-test (unpaired, two-tailed, **P* < 0.05).

***Fig. S6: Biophysical characterization of purified Bcl-xL recombinant proteins.***

**a** 6xHis-Bcl-xL, 6xHis-Bcl-xL^ΔTMD^ and 6xHis-Bcl-xL^K87D^ recombinant proteins were purified as described in the Material and Methods section. 2 µg of proteins were then subjected to SDS-PAGE and the gel was stained with Coomasie blue. **b** Far‐UV CD spectra analysis of 6xHis-Bcl-xL and 6xHis-Bcl-xL^ΔTMD^ and 6xHis-Bcl-xL^K87D^ mutant derivatives. Experiments, carried out at 20 °C (15 μm; 250 μL; 5 mM MOPS, pH: 7.5; 5 mM NaCl), were normalized for protein concentration; [θ]: molar ellipticity. The differences seen are attributed to the effect of the mutations on the helical content of Bcl‐xL (*N* = 4). Representative spectra are shown following smoothing (5FFT). **c** Thermal ramping denaturation curves (15–90 °C) obtained by monitoring ellipticity at 222 nm, by far‐UV CD, while heating the protein samples (15 μm; 250 μL; 5 mM MOPS, pH: 7.5; 5 mM NaCl) at 1 °C· min^−1^ (*N* = 4). A representative experiment is shown following smoothing (15FFT) and normalization of all curves at the first data point.

***Fig. S7: Examination of HeLa cells treated with staurosporine***

**a** This panel displays the single-cell Ca^2+^ signals evoked by STS (0.5 μM) for which the average response was shown in Fig. 7e. Ca^2+^ signals were obtained from Fura-2-loaded wild type HeLa transfected with empty vector (pCMV24-mCherry; black) Bcl-xL (pCMV24-3xFLAG-Bcl-xL-P2A-mCherry; green) or Bcl-xL^K87D^-coding vector (pCMV24-3xFLAG-Bcl-xL^K87D^-P2A-mCherry; red). **b** Areas under the curve calculated from the calcium measurements traces shown in **a**. Contrary to Fig. 7f, the data are represented here according to each independent experiment. For each condition, 1 to 2 independent wells obtained from 4 different transfections on different days were monitored. Each point represents one individual cell. Statistically significant differences were determined using a two-way ANOVA (*P < 0.05). **c** Wild type HeLa transiently overexpressing 3xFLAG-Bcl-xL or 3xFLAG-Bcl-xL^K87D^ were treated with 0.25 or 0.5 µM STS for six hours. The samples were analyzed via western blot, utilizing each time independently transfected and treated cells. Representative western blots assessing uncleaved and cleaved PARP as well as total Bcl-xL and β-actin. This panel is the uncropped version of the picture shown in Fig. 7g.

***Fig. S8: Bcl-2 expression compensates*** Bcl-xL knockdown.

MDA-MB-231 cells were transfected with either a siRNA targeting Bcl-xL (siBcl-xL) or a non-target siRNA (siCtrl). 48 hours later, the cells were lysed and proteins were analyzed via western blot (IB: immunoblot). **a** Representative western blots assessing Bcl-2 and vinculin. 20 µg of MDA-MB-231 and 10 µg of OCI-LY-1 cell lysates were loaded. Quantifications are shown in **b**. Data are represented as mean ± SD (*N* = 4), each point represents one experiment with independent transfections. Statistically significant differences were determined using a *t*-test (unpaired, two-tailed, **P* < 0.05).

***Fig. S9: Bcl-xL overexpression does not alter ER Ca^2+^-store content in DT40 cells.***

Ca^2+^ measurements obtained from Fluo-4-loaded DT40 cells transfected with an empty vector (black) or a 3xFLAG-Bcl-xL-P2A-mCherry plasmid (green). ER Ca^2+^ loading was determined by measuring the response to thapsigargin (2 µM) after addition of the extracellular Ca^2+^ buffer EGTA (3 mM). Ionomycin (5 µM) diluted in 250 mM CaCl_2_ was added at the end of the experiment (iono.) to trigger a high Ca^2+^ release and validate the loading with Fluo-4. **a and b** The Ca^2+^ traces represent the average response ± SEM of every cells in one well containing about 20-30 cells. For each condition, 6 to 9 independent wells obtained from 3 different transfections were monitored. **c** Areas under the curve were calculated from the Ca^2+^ traces. Data represent mean ± SD of every wells (*N* = 6 to 9), each point represents one well. Statistically significant differences were determined using a *t*-test (unpaired, two-tailed, **P* < 0.05).
