# Supplementary figures and images for: Bcl-xL acts as an inhibitor of IP3R channels, thereby antagonizing Ca2+-driven apoptosis
Source: Cell Death Differ. 2021 Nov 8;29(4):788–805. doi: 10.1038/s41418-021-00894-w (PMC8990011; doi:10.1038/s41418-021-00894-w)

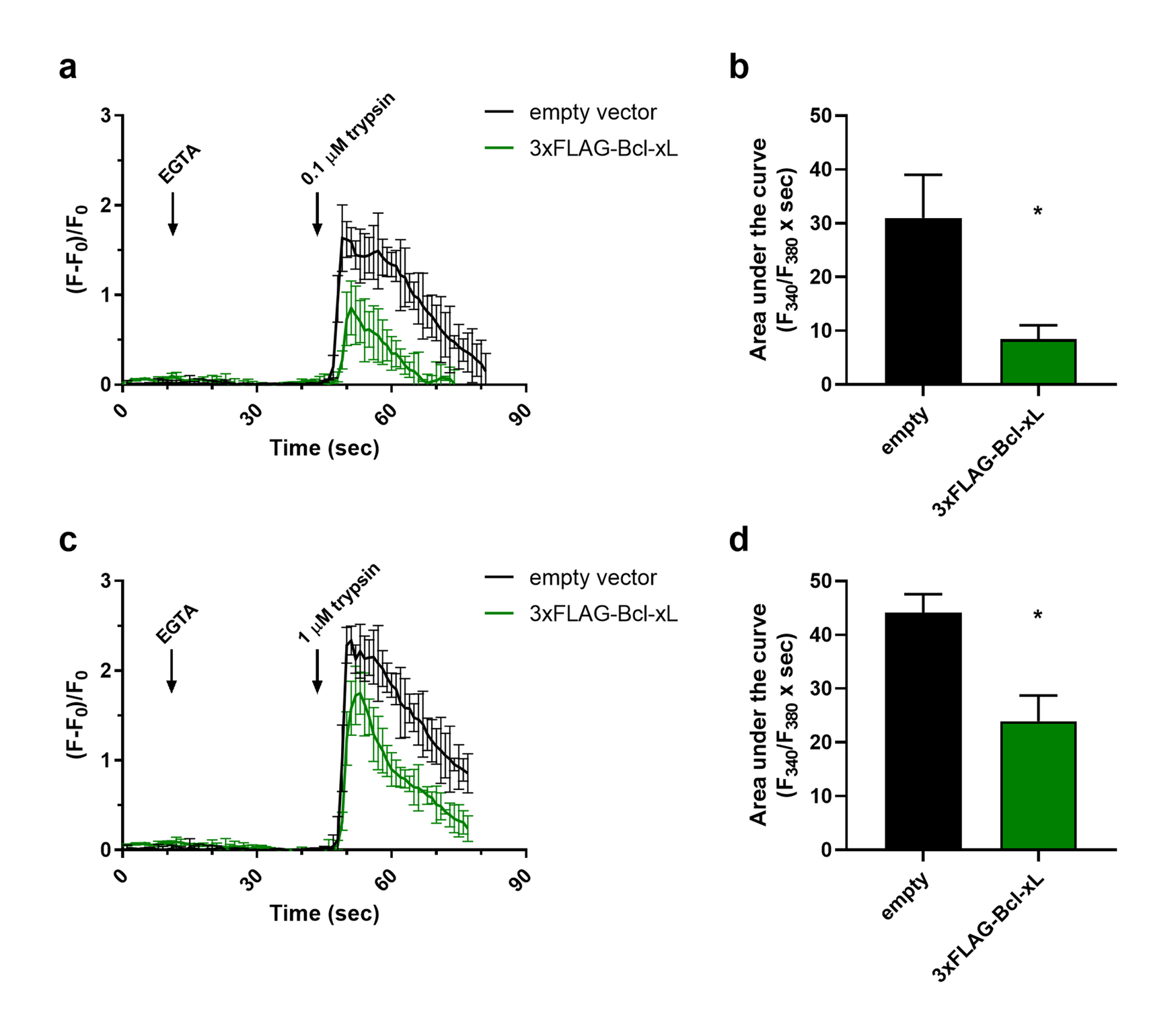

Supplement: Supplementary file 2 — Supplemental Figure 1 [file 41418_2021_894_MOESM2_ESM.tif]

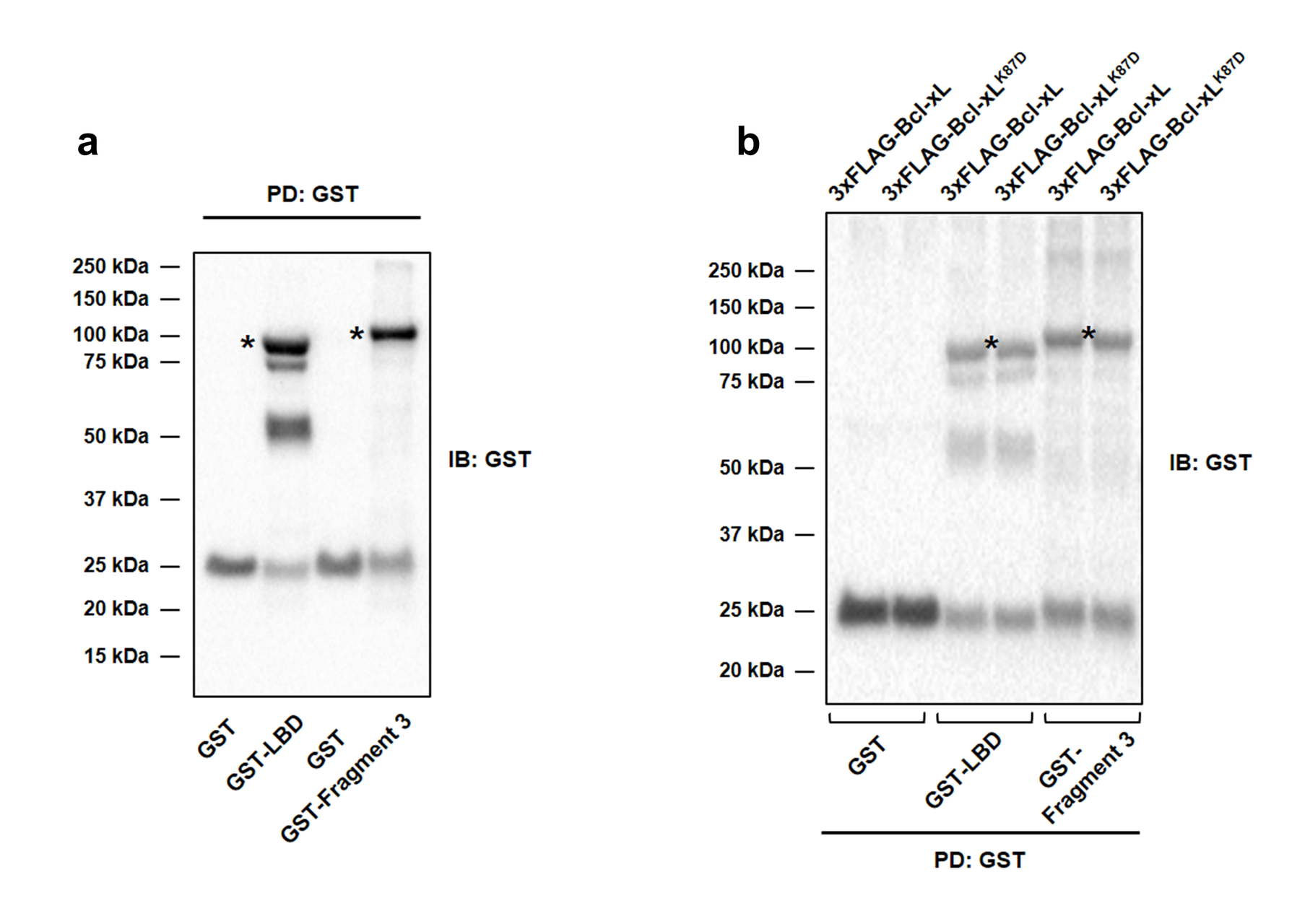

Supplement: Supplementary file 3 — Supplemental Figure 2 [file 41418_2021_894_MOESM3_ESM.tif]

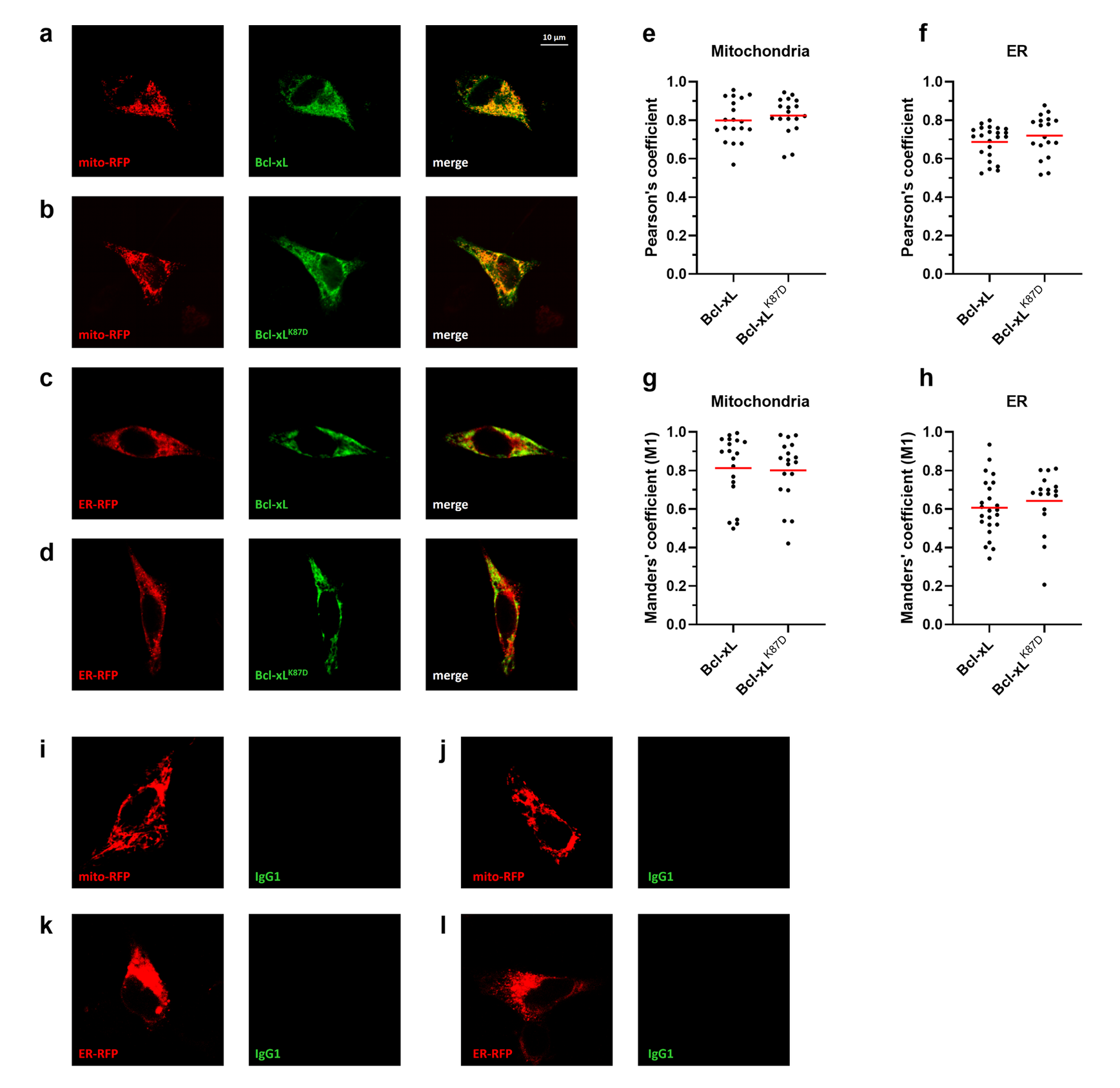

Supplement: Supplementary file 4 — Supplemental Figure 3 [file 41418_2021_894_MOESM4_ESM.tif]

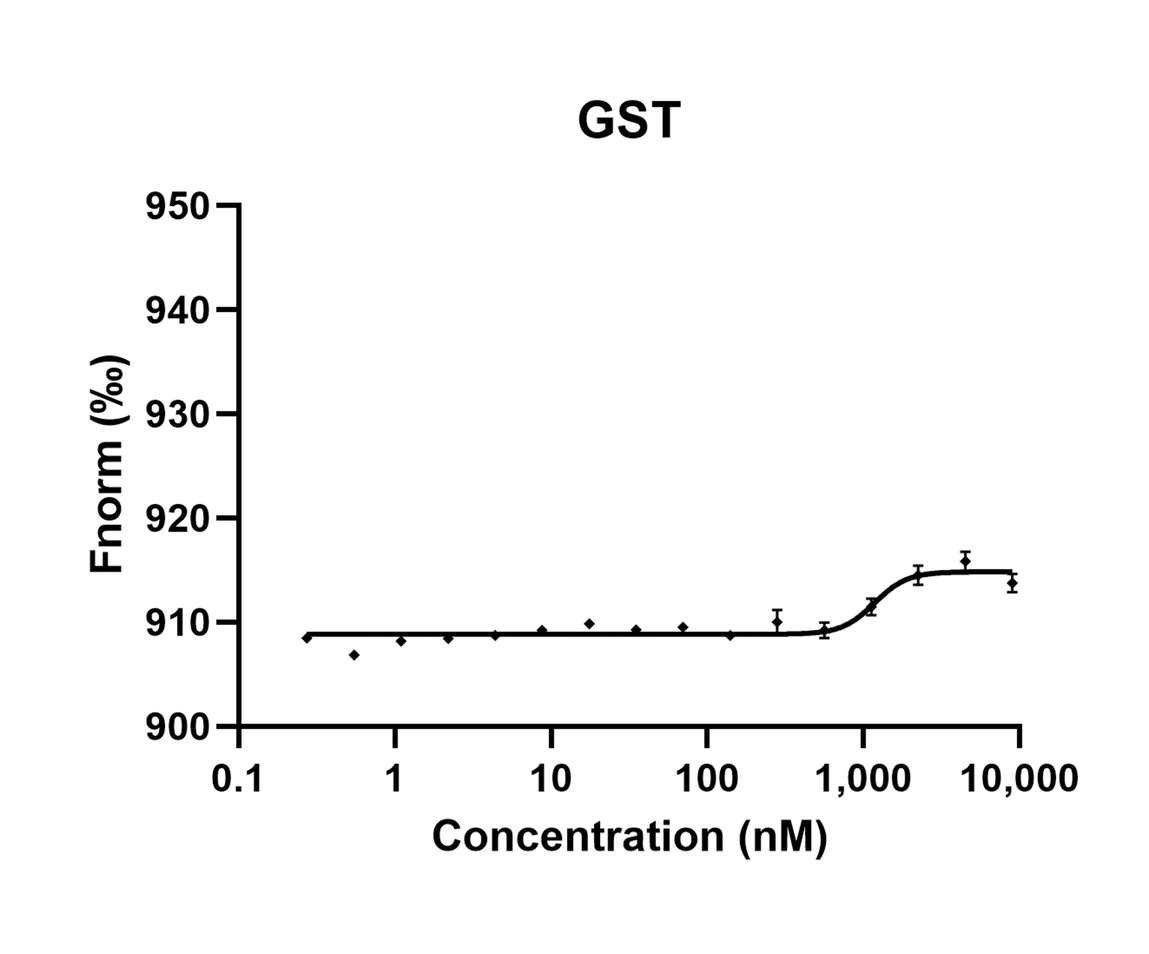

Supplement: Supplementary file 5 — Supplemental Figure 4 [file 41418_2021_894_MOESM5_ESM.tif]

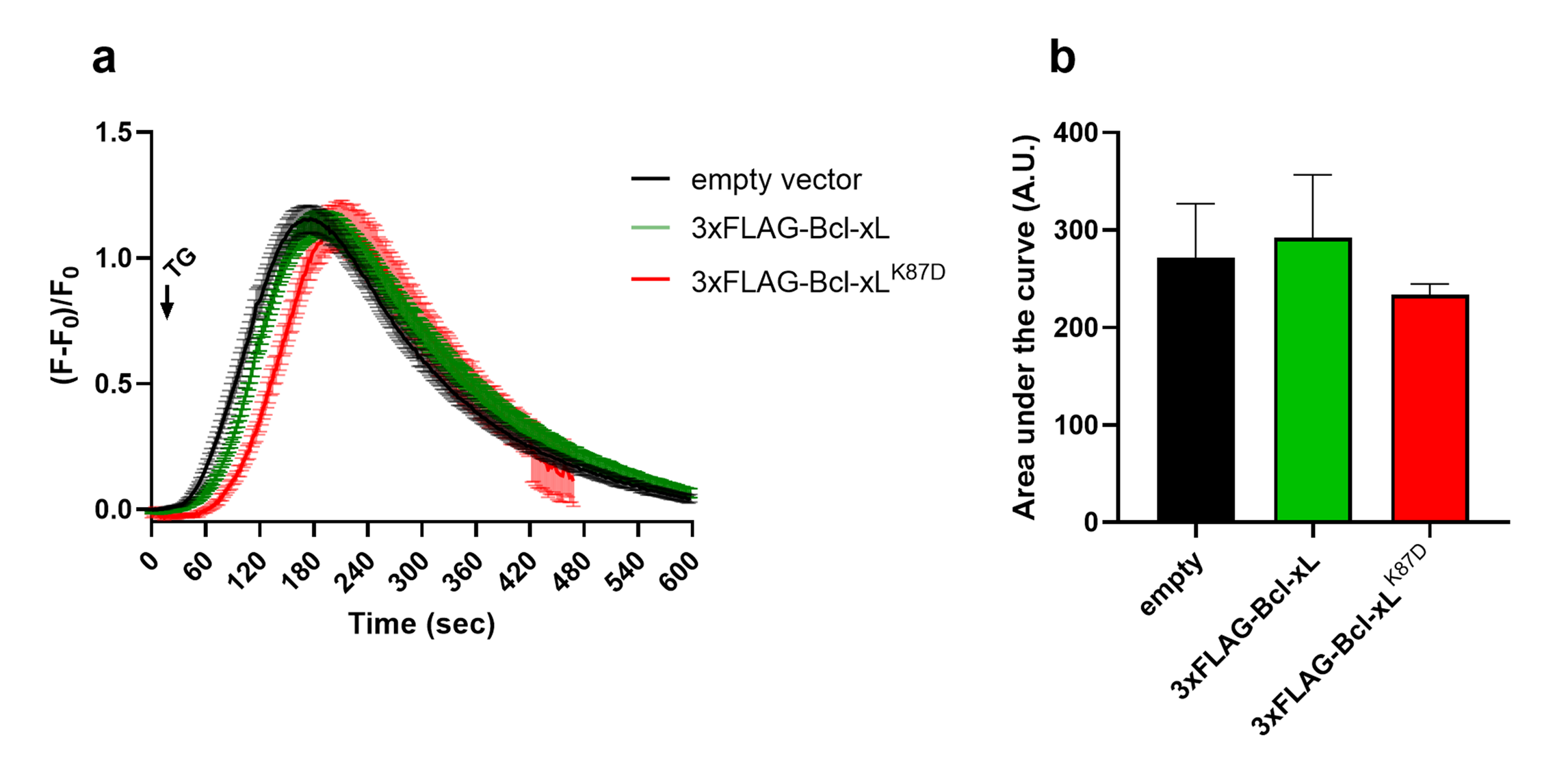

Supplement: Supplementary file 6 — Supplemental Figure 5 [file 41418_2021_894_MOESM6_ESM.tif]

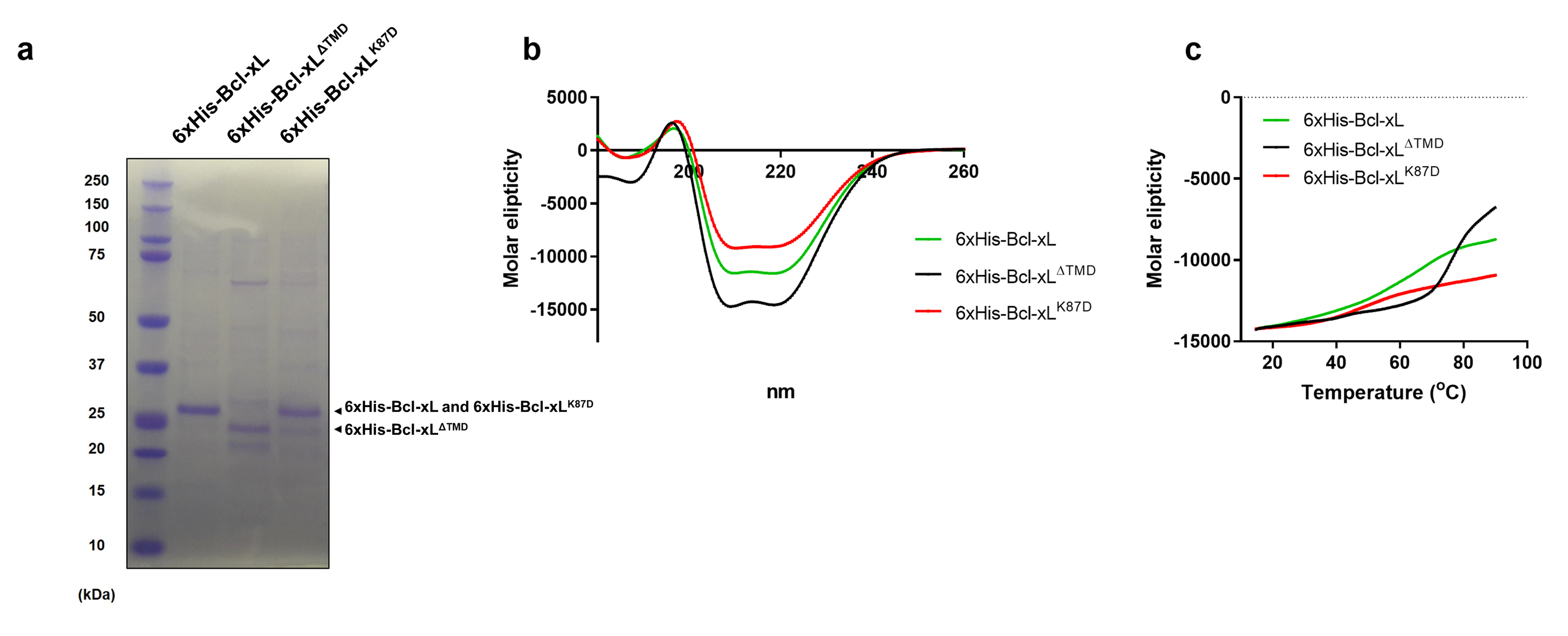

Supplement: Supplementary file 7 — Supplemental Figure 6 [file 41418_2021_894_MOESM7_ESM.tif]

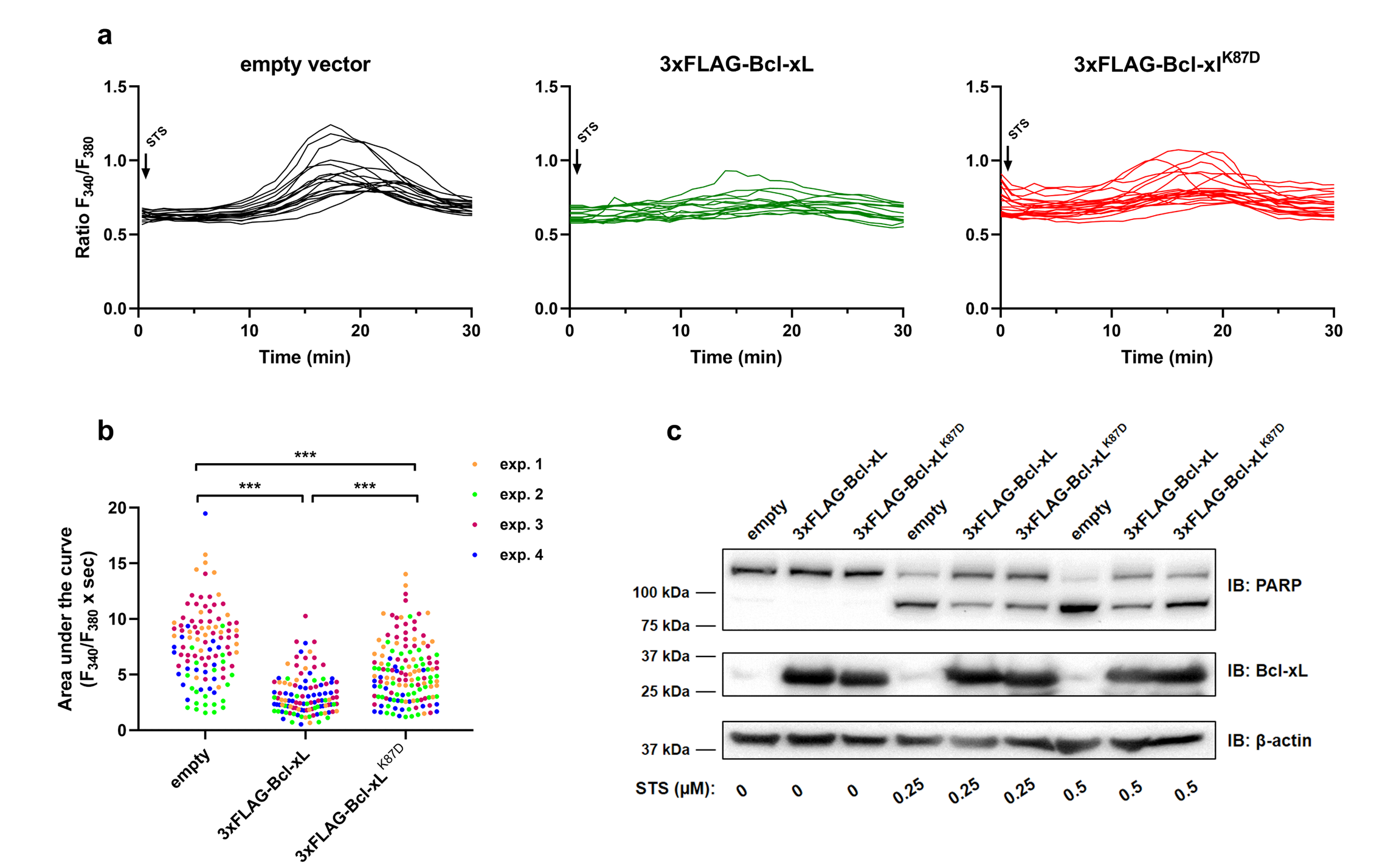

Supplement: Supplementary file 8 — Supplemental Figure 7 [file 41418_2021_894_MOESM8_ESM.tif]

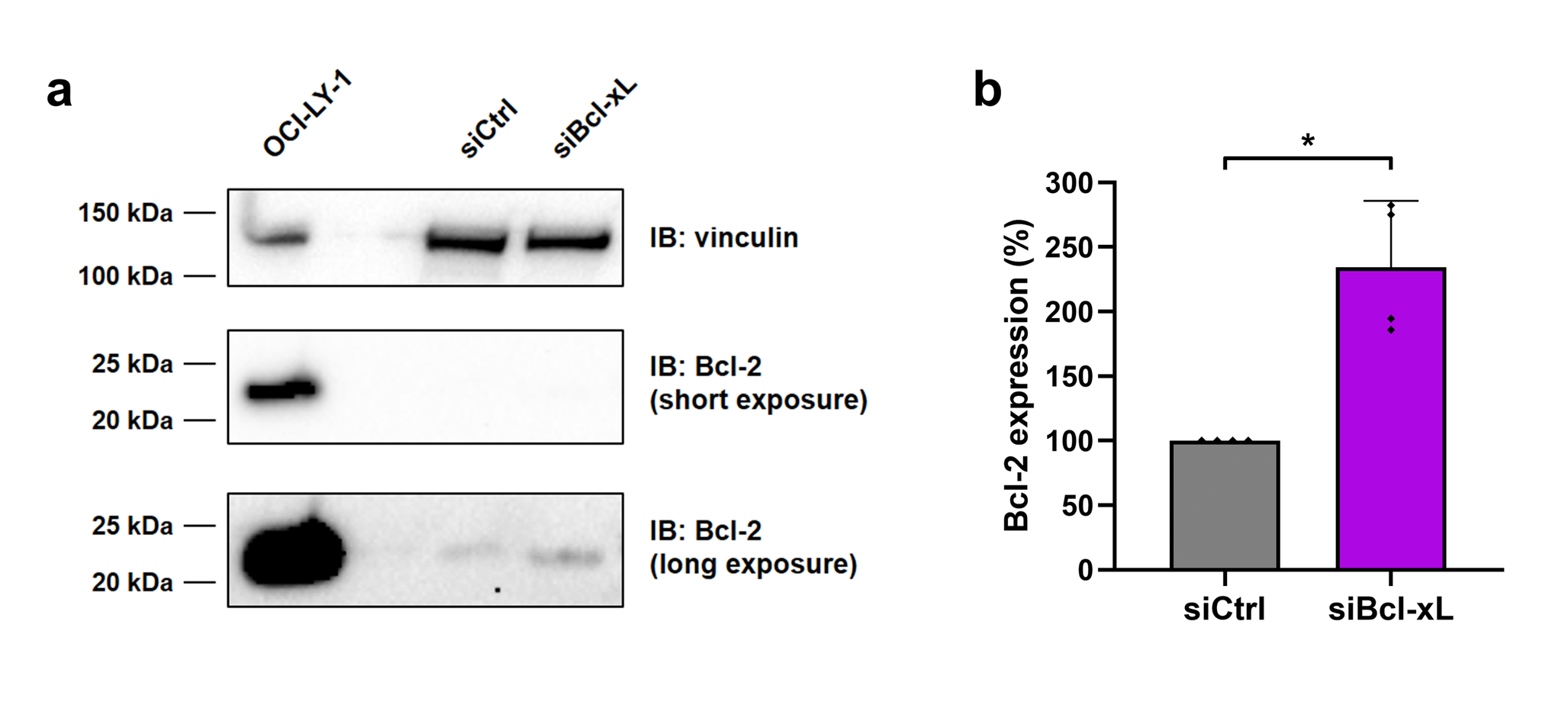

Supplement: Supplementary file 9 — Supplemental Figure 8 [file 41418_2021_894_MOESM9_ESM.tif]

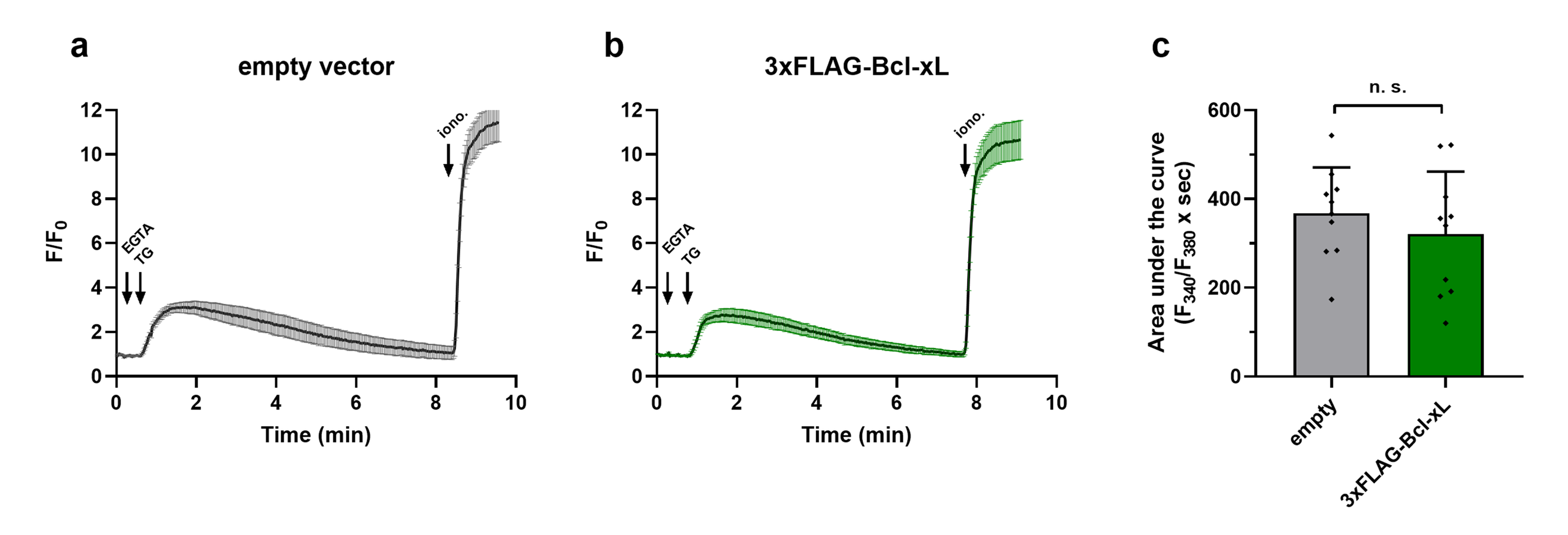

Supplement: Supplementary file 10 — Supplemental Figure 9 [file 41418_2021_894_MOESM10_ESM.tif]
